# Magnetic field assisted high capacity durable Li-ion battery using magnetic α-Fe_2_O_3_ nanoparticles decorated expired drug derived N-doped carbon anode

**DOI:** 10.1038/s41598-020-67042-1

**Published:** 2020-06-19

**Authors:** Dipsikha Ganguly, Ajay Piriya V.S., Anamika Ghosh, Sundara Ramaprabhu

**Affiliations:** Alternative Energy and Nanotechnology Laboratory, Department of Physics, Indian Institute of Technology Madras, Chennai, 600036 India

**Keywords:** Nanoscale materials, Energy storage

## Abstract

We have synthesized a novel ferromagnetic material by coating α-Fe_2_O_3_ nanoparticles with N-doped carbon matrix using a simple combustion method. Expired paracetamol drugs are used as nitrogen and carbon source. This α-Fe_2_O_3_/NC shows ferromagnetic property due to the incorporation of oxygen defects. When used as the Li-ion battery anode, α-Fe_2_O_3_/NC shows higher capacity compared to commercial α-Fe_2_O_3_ due to the occurrence of both intercalation and conversion reaction. Further, application of magnetic field at the anode of the freshly assembled cell at the first charge-discharge cycle, results in ~two-fold enhancement in specific capacity. For the cycled cell also, increase in the capacity from 80 mAh. g^−1^ to 150 mAh. g^−1^ at 5 A. g^−1^ is observed during the application of magnetic field at the 501^st^ charging cycle. This improved performance is attributed to the field-dependent enhancement of diffusion and convection due to the magnetohydrodynamic effect. Further, application of the magnetic field at 1001^st^, 1501^st^ and 1751^st^ charging cycles shows improved LIB performance. We can show that not only the magnetic field, magnetic properties of the anode α-Fe_2_O_3_/NC also play a crucial role in influencing the battery performance. Moreover, utilization of expired drug helps in dramatically reducing pollution caused by its disposal.

## Introduction

The depletion of fossil fuels and the concomitant increase of CO_2_ effluence have stimulated the need for alternative energy technologies for the future society. One of the promising solutions is to shift from the existing fuel-powered vehicles to battery-powered ones. However, this demands high capacity lithium-ion batteries (LIBs), which has engendered the need for the research of alternative anode materials^1–4^. Amongst the metal oxides, α-Fe_2_O_3_ is widely studied due to its high theoretical capacity, excellent catalytic properties, electrochemical and thermal stability^3,5–8^. During Li^+^ ion insertion in bulk α-Fe_2_O_3_, iron atoms jump from tetrahedral sites to octahedral sites and result in face centred cubic Fe_3_O_4_ structure and produces Fe and Li_2_O matrix^9^. But this mechanism does not hold good for its nano counterpart^10^. The reduced particle size of α-Fe_2_O_3_ nanoparticles results in intercalation without the change in the crystal structure, thereby facilitates better Li^+^ ion storage and durability in α-Fe_2_O_3_ anode^10,11^.

But, the main drawback of α-Fe_2_O_3_ is its large volume expansion which leads to the low durability and capacity fading over the cycles. Incorporation of high surface area porous carbon matrix helps to accommodate the volume expansion during lithiation/delithiation process^5,7,12^. Compared to pure carbon matrix, N-doping on carbon helps in better interaction of Li and N doped carbon due to the higher electronegativity of nitrogen and change in the electron density near carbon atoms. This eventually lead to better stability of LIBs^1,12,13^.

Despite these, lithium deposition at anode and increasing concentration overpotential cause capacity fading during long-term cycling^5,14–18^. To reduce this, Billaud *et al*. have reported magnetic Fe_3_O_4_ nanoparticles grafted aligned graphite anode in the presence of a magnetic field, but its limited for low-capacity applications^19^. In another report, Shen *et al*. have demonstrated that better stability could be achieved in lithium metal batteries by suppressing dendrite formation employing a magnetic field of 3500 Gauss, but this is also limited to alkali metal batteries^20^. Recently, Zhang *et al*. have shown that voltage-controlled magnetic switching is an important phenomenon for α-Fe_2_O_3_ due to the change in magnetization during lithiation-delithiation state^21^. With the decrease in the discharge potential, an increase in the saturation magnetization is observed with the change in the coercivity. This was ascribed to the Fe_2_O_3_ to Fe conversion with the enhanced lithiation state, whereas during charging, magnetization is decreased owing to its antiferromagnetic nature and hence can be demagnetized easily. Recently, Biswas *et al*. have further revealed that Na-ion supercapacitor performance can be increased with the application of magnetic field due to the occurrence of Fe^3+^ ions in NaFePO_4_^22^.

Herein, we propose ferromagnetic α-Fe_2_O_3_/NC anode for LIBs. Ferromagnetism is introduced due to the incorporation of oxygen defects. Further, influence on the battery performance with magnetic α-Fe_2_O_3_/NC anode is explored in presence of an external magnetic field at the anode. Application of magnetic field for the initial charge-discharge cycle results in higher capacity and longer cycle life due to the enhanced lithiation/delithiation and lower electrochemical resistance. Further, our work shows that the application of the magnetic field can also increase the capacity and cycle life of the cycled cells. We have also demonstrated that the magnetic field applied during single charging of cell after 500 cycles, the capacity can be enhanced to 45% at the end of 1000 cycles and are stable upto 2000 cycles. Also, with the application of a magnetic field for a few charge-discharge cycles after cycling, 100% capacity retention of LIB is achieved. The capacity enhancement of cycled cells with this technique can avoid tedious and expensive recycling process^23,24^. To the best of our knowledge, enhancing the capacity and durability of LIBs with magnetic anode and magnetic field for cycled and fresh cells with the utilization of the expired drugs are not reported.

## Results

### Structural and morphological studies of α-Fe_2_O_3_/NC anode

**Figure 1:**
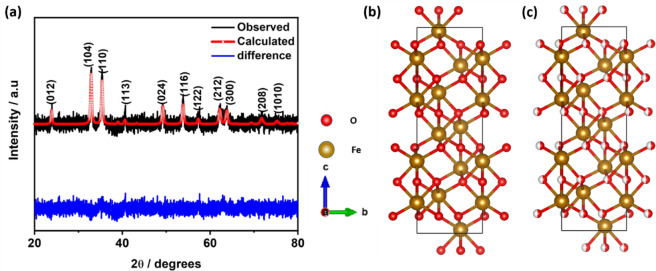
(**a)** Rietveld fitted X-ray diffraction pattern for α-Fe_2_O_3_/NC, Simulated structure of **(b)** α-Fe_2_O_3_ and **(c)** α-Fe_2_O_3_/NC.

The X-ray diffraction (XRD) study of α-Fe_2_O_3_/NC shows the presence of (012), (104), (110), (113), (024), (116), (122), (212), (300), (208) and (1010) planes which are the fingerprint of α-Fe_2_O_3_ (Fig. 1a) (ICDD # 98-000-6273) phase.

The crystallite size calculated from the lower angle peaks using Debye-Scherrer formula (equation S1) is ~20.9 nm. Shifting of the XRD peaks to lower angle (Table S1) indicates the change in the crystal parameters due to the presence of defects. Detailed Rietveld analysis was further done to probe the changes in the crystal structure and anion-cation distribution (Table 1). Along with the observable shift in the crystal lattice parameters, occupancy of the oxygen atoms changes from 1 to 0.499 confirms the presence of oxygen vacancies and change in cationic-anionic distribution as evident from X-ray photoelectron spectroscopy analysis (Fig. S1). The simulated crystal structures of pure α-Fe_2_O_3_ and α-Fe_2_O_3_/NC obtained from Rietveld analysis are shown in Fig. 1(b,c). In addition, due to the higher content of α-Fe_2_O_3_ in α-Fe_2_O_3_/NC, and presence of disordered carbon, carbon planes are not prominent in XRD. In order to characterize the carbon XPS, Thermogravimetric analysis (TGA) and Raman analysis are shown in Figures S1–S3. Due to the Presence of these vacancies room temperature ferromagnetism is observed in α-Fe_2_O_3_/NC (Fig. S4**)**.

**Table 1:** Rietveld analysis parameter comparison of α-Fe_2_O_3_ and α-Fe_2_O_3_/NC.

crystal lattice parameter comparison of α-Fe_2_O_3_ and α-Fe_2_O_3_/NC						
	a (Å)	b (Å)	c (Å)	α	Β	γ
**α-Fe**_**2**_**O**_**3**_	5.038	5.038	13.77	90°	90°	120°
**α-Fe**_**2**_**O**_**3**_**/NC**	5.041	5.041	13.76	90°	90°	120°
**Atomic positional parameter of α-Fe**_**2**_**O**_**3**_						
	**x**	**y**	**z**	**Occupancy**	**Site**	**Symmetry**
**O**	0.3051	0	0.25	1	18e	0.2
**Fe**	0	0	0.3557	1	12c	3.0
**Atomic positional parameter of α-Fe**_**2**_**O**_**3**_**/NC**						
	**x**	**y**	**z**	**Occupancy**	**Site**	**Symmetry**
**O**	0.3051	0	0.25	0.499	18e	0.2
**Fe**	0	0	0.3551	1.001	12c	3.0

**Figure 2:**
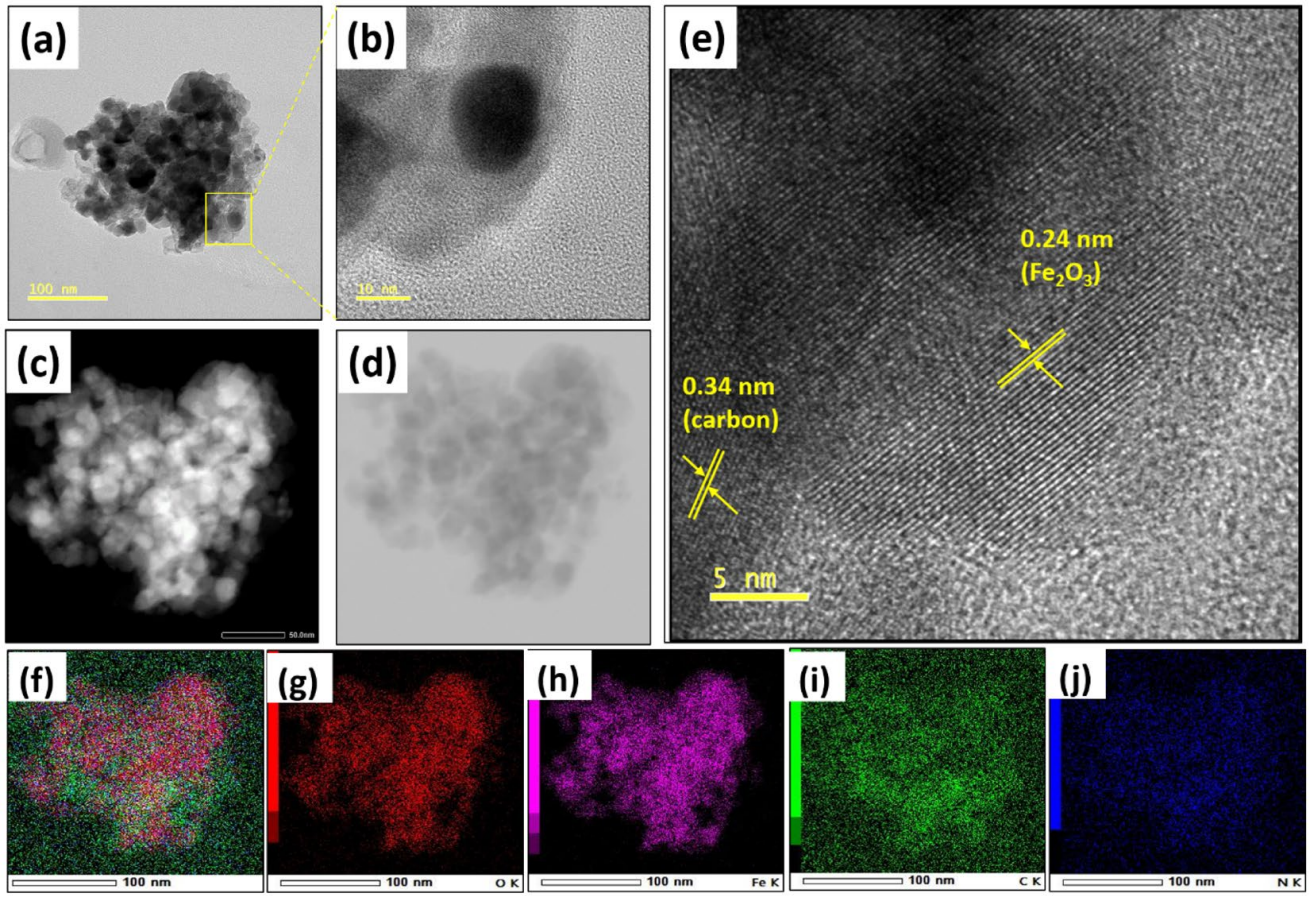
(**a,b)** HRTEM images, **(c,d)** STEM-HAADF images **(e)** lattice fringes from HRTEM analysis **(f-j)** elemental mapping of α-Fe_2_O_3_/NC.

High resolution transmission electron micrograph (HRTEM) shows the formation of nanosized α-Fe_2_O_3_ particles coated with N-doped carbon (Fig. 2(a,b)). Z-contrast image shown in Fig. 2(c,d) obtained from high-angle annular dark-field scanning transmission electron microscopy (HAADF-STEM), confirms carbon coating over α-Fe_2_O_3_ nanoparticles where Fe atom with high Z value appears to be darker compared to the lighter N or C atoms. Average size of the particle is estimated ~20 nm, which is in good agreement with XRD studies. Lattice spacings of α-Fe_2_O_3_ particle and the carbon coating on of α-Fe_2_O_3_ calculated from HRTEM lattice fringes are 0.24 nm and ~0.34 nm, correspond to α-Fe_2_O_3_ (110) planes and C (002) planes respectively (Fig. 2e). Elemental mapping in Fig. 2(f–j) shows the distribution of Fe, C, O and N over the specified region. High surface area (179 m^2^. g^−1^) and porous nature of α-Fe_2_O_3_/NC are explained by field emission scanning electron microscopy (FESEM) and Brunauer-Emmett-Teller (BET) analysis in Figures S5–S6.

### Electrochemical response of α-Fe_2_O_3_/NC anode

**Figure 3:**
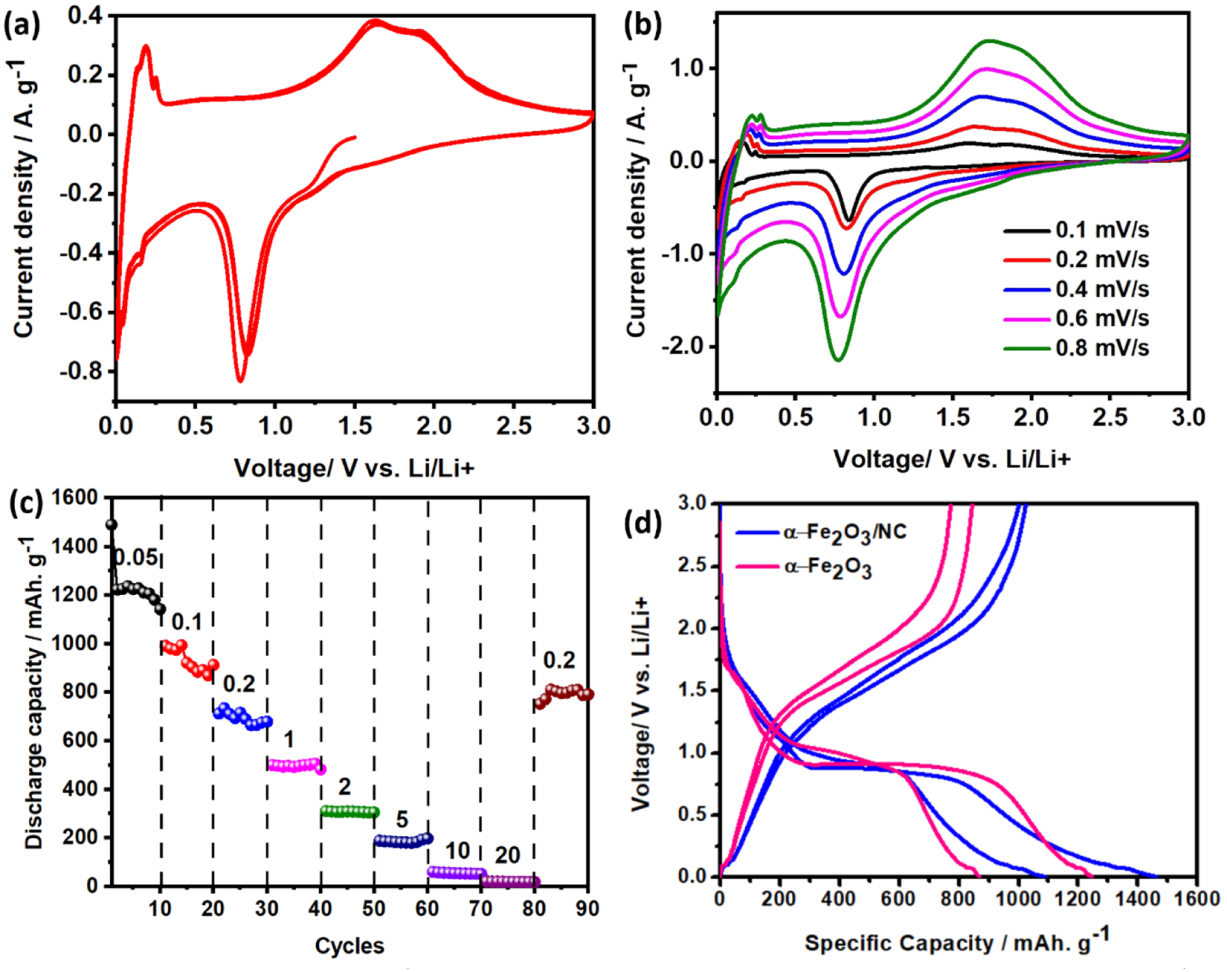
(**a)** CV at 0.1 mV.s^−1^ for 3 cycles **(b)** scan rate variation from 0.1–0.8 mV. s^−1^ **(c)** rate capability of α-Fe_2_O_3_/NC anode (in A. g^−1^ units), and **(d)** Comparison of capacity of α-Fe_2_O_3_ and α-Fe_2_O_3_/NC at 50 mA. g^−1^.

Figure 3(a) shows the cyclic voltammetry (CV) curves of assembled coin cell using α-Fe_2_O_3_/NC as anode for the 3 cycles at a scan rate of 0.1 mV. s^−1^. Anodic peaks at 1.62 V and 1.87 V are ascribed to the oxidation of Fe° to Fe^2+^ and further oxidation to Fe^3+^. A sharp cathodic peak at 0.78 V is observed in the 1^st^ cycle, further shifts to 0.82 V in subsequent cycles confirming the solid electrolyte interphase (SEI) layer formation due to the decomposition of the electrolyte and reduction of Fe^3+^ to Fe° species^8^. Another cathodic peak located at 1.68 V is ascribed to initial lithiation of α-Fe_2_O_3_/NC in hexagonal phase with a minor peak at ~1.2 V, which arises due to the phase transition from hexagonal to cubic phase^8^. The cathodic and anodic peaks in the lower voltage region (0–0.3 V) attribute to the successful intercalation and deintercalation of lithium ion into N doped porous carbon matrix respectively^25,26^. Electrochemical stability of the anode after SEI formation can be confirmed from the overlapped CV curves obtained in 2^nd^ and 3^rd^ cycles (Fig. 3a). The increasing trend of current with increasing scan rate confirms it as a diffusion-controlled phenomenon (Fig. 3b).

Rate capability study of the assembled cell cycled at different current densities (0.05–20 A. g^−1^) shows the recovery of 100% capacity at 0.2 A. g^−1^ after extensive cycling (Fig. 3c**)**, indicating α-Fe_2_O_3_/NC as high rate capable anode. Figure 3d shows the 1^st^ and 2^nd^ cycle charge-discharge profile of commercial α-Fe_2_O_3_ and α-Fe_2_O_3_/NC at 50 mA. g^−1^. The first discharge cycle of α-Fe_2_O_3_/NC anode shows four different regions correspond to the different types of Li^+^ ion interactions with α-Fe_2_O_3_/NC (Fig. 3d). Initially, voltage is rapidly decreased from 3 V to 1.68 V, which corresponds to the lithiation in hexagonal α-Fe_2_O_3_ phase. Another slope at ~1.2 V is assigned to the hexagonal to cubic phase transition. A long plateau region observed at 0.78 V delivering a specific capacity of 800 mAh. g^−1^ corresponds to Fe^3+^ to Fe° transition. Further, the voltage profile is dropped smoothly to 0.005 V with a specific capacity of 1500 mAh. g^−1^. The sloping region below 0.78 V corresponds to the formation of the SEI layer due to decomposition of electrolyte and interaction of Li^+^ ions with N doped carbon matrix. Charge discharge profiles at different current densities (0.1–20 A. g^−1^) and 1^st^, 10^th^, 50^th^ and 250^th^ cycles at a fixed current density (0.4 A. g^−1^) are shown in Figure S7. *Ex-situ* FESEM shows the gradual formation of the SEI layer at different discharge voltages (Fig. S8**)**. Discharge profile of α-Fe_2_O_3_/NC, commercial α-Fe_2_O_3_ with same mass loading at a current density of 50 mA. g^−1^ shown in Fig. 3d results in a higher specific capacity of the α-Fe_2_O_3_/NC (1500 mAh. g^−1^) compared to commercial α-Fe_2_O_3_ (1250 mAh. g^−1^). Presence of porous N-doped carbon matrix and reduced particle size of α-Fe_2_O_3_/NC result in more lithium utilization concomitantly increases the capacity.

Ideally, insertion of Li^+^ ion in α-Fe_2_O_3_ induces perpendicular stress to c-axis resulting in a structural transformation from hcp to ccp^9^. Due to the reduction in particle size to nano regime and in the presence of oxygen vacancies, intercalation of Li^+^ ions into the empty octahedral sites is more probable instead of growing new phases^11^. Further, the existence of mixed phases observed for deep discharge can be explained through Eqs. (1–3):

1 $${\rm{\alpha }}-{{\rm{Fe}}}_{2}{{\rm{O}}}_{3}\to {{\rm{Li}}}_{{\rm{x}}}{{\rm{Fe}}}_{2}{{\rm{O}}}_{3}$$

2 $${\rm{\alpha }}-{{\rm{Fe}}}_{2}{{\rm{O}}}_{3}\to {{\rm{Li}}}_{{\rm{x}}}{{\rm{Fe}}}_{2}{{\rm{O}}}_{3}+{{\rm{Li}}}_{{\rm{y}}}{{\rm{Fe}}}_{3}{{\rm{O}}}_{4}$$

3 $${{\rm{Li}}}_{{\rm{x}}}{{\rm{Fe}}}_{2}{{\rm{O}}}_{3}\to {\rm{Fe}}+{{\rm{Li}}}_{2}{\rm{O}}$$

### Influence of magnetic field on LIB performance with α-Fe_2_O_3_/NC anode

Magnetic field effect on the LIB performance is first probed using CV studies. Magnetic field (0.15 T) is applied at the anode side of the coin cell (already tested for previous CV measurement).

**Figure 4:**
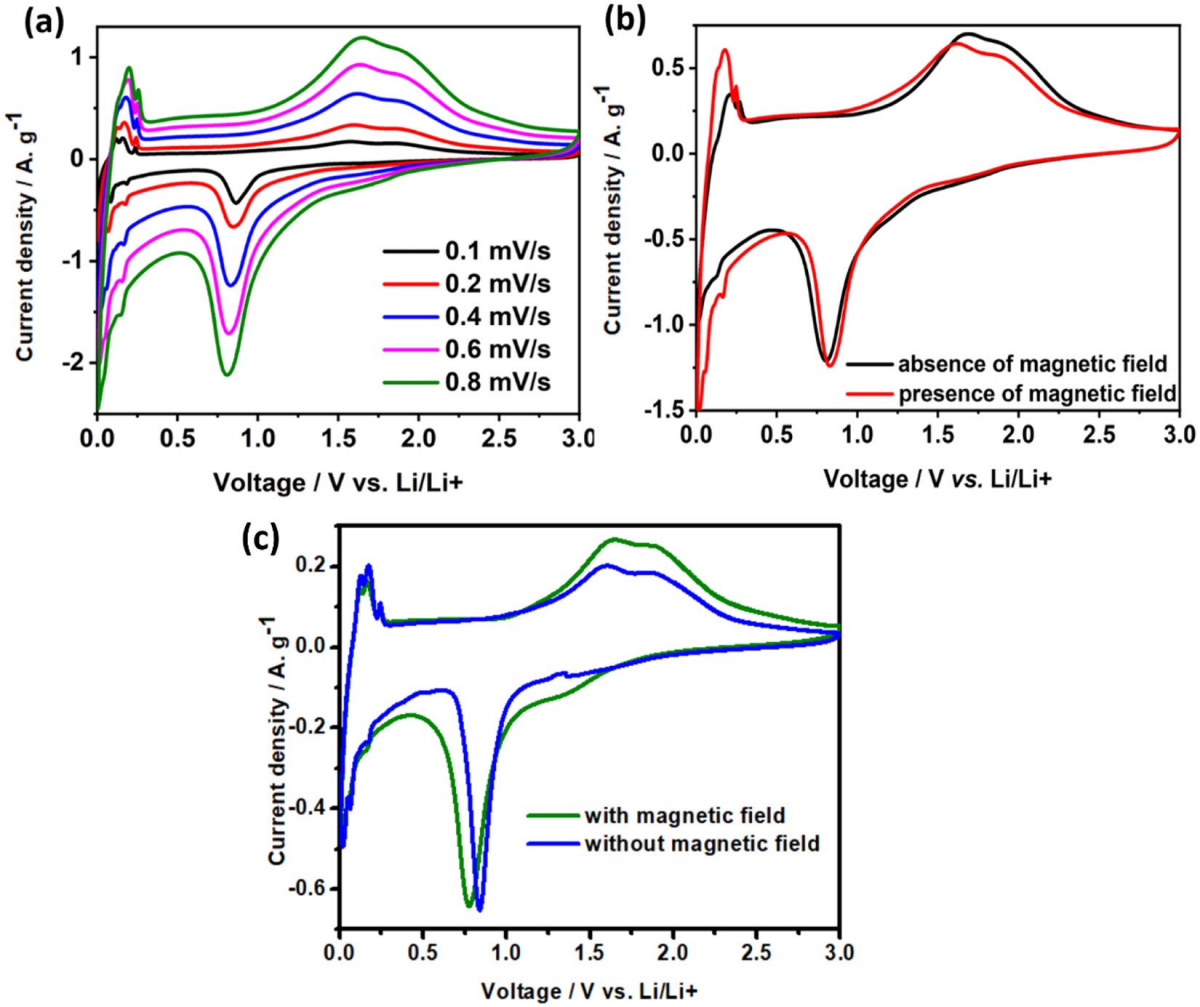
(**a**) Scan rate variation of α-Fe_2_O_3_/NC in the presence of the magnetic field; Comparison of CV (**b**) for the same cell (at 0.4 mV. s^−1^) and (**c**) fresh cell (at 0.1 mV. s^−1^) in the presence and absence of magnetic field.

As seen in Fig. 4(a,b), significant changes in the peak current and peak areas are observed in the presence of the magnetic field. The current corresponding to the cathodic and anodic peaks at 0–0.3 V increases in the presence of the magnetic field which signifies better intercalation-deintercalation of Li^+^ ions onto the carbon matrix^25,26^. Along with the enhancement of peak currents in the lower voltage region, change in the anodic peak ratios of 1.62 V, and 1.87 V signifies the existence of both hcp and ccp structures. Prominent splitting of the anodic peaks of 1.62 V and 1.87 V and enhanced peak area under 1.62 V compared to 1.87 V on the application of magnetic field attribute to the better kinetics of Fe° to Fe^2+^ oxidation compared to Fe^2+^ to Fe^3+^ oxidation. Besides, Li^+^ ion intercalation peak at 0.82 V is enhanced on the application of the magnetic field. To have a better insight into the role of the magnetic field on the battery performance, diffusion coefficients (D_o_) of Li^+^ ions are calculated using Randles-Sevcik equation (equation S2**)** (Table 2). Increase in D_o_ of the cathodic peak at 0.82 V from 3.89 × 10^−14^ cm^2^. s^−1^ to 4.32 × 10^−14^ cm^2^. s^−1^ asserts better intercalation of Li^+^ ion into α-Fe_2_O_3_/NC. For the 0.5–3 V region, D_o_ reduces for anodic peaks validating the intercalation to be the governing mechanism for LIB. The area under the peaks (1.62 V and 1.87 V) confirms the conversion of Fe^0^ to Fe^2+^, which is more feasible compared to Fe^2+^ to Fe^3+^ transition due to the non-magnetic nature of Fe^2+^ ion^27^.

**Table 2:** Diffusion coefficients obtained from CV using the Randles-Sevcik equation for different anodic and cathodic peaks.

Peak voltage	Diffusion coefficient (Do) of Anodic peak (cm^2^. s^−1^)				
	(0.11 V)	(0.17 V)	(0.24 V)	(1.62 V)	(1.87 V)
α-Fe_2_O_3_/NC	0.99 × 10^−14^	1.26 × 10^−14^	0.86 × 10^−14^	2.0 × 10^−14^	1.69 × 10^−14^
α-Fe_2_O_3_/NC (with magnetic field)	1.09 × 10^−14^	1.49 × 10^−14^	0.99 × 10^−14^	1.97 × 10^−14^	1.49 × 10^−14^
**Peak voltage**	**Diffusion coefficient (Do) of cathodic peak (cm**^**2**^**. s**^−1^**)**				
	**(0.047 V)**	**(0.083 V)**	**(0.185 V)**	**(0.82 V)**	**(1.68 V)**
α-Fe_2_O_3_/NC	2.45 × 10^−14^	2.37 × 10^−14^	2.69 × 10^−14^	3.89 × 10^−14^	0.55 × 10^−14^
α-Fe_2_O_3_/NC (with magnetic field)	4.89 × 10^−14^	4.45 × 10^−14^	3.15 × 10^−14^	4.32 × 10^−14^	0.52 × 10^−14^

Though the diffusion coefficient of Li^+^ ion is improved, since the same cell has been used for the scan rate variation without magnetic field, the difference in the current and peak splitting is not very prominent. Hence, a new cell is assembled and magnetic field is applied from the initial CV cycle. Figure 4c shows the comparison of the CV profile of the two new cells with and without magnetic field at a scan rate of 0.1 mV. s^−1.^ Drastic change in the currents of cathodic peak (0.8 V) and anodic peaks (1.62 V and 1.9 V) is observed. To have a better insight, area under the redox peaks of α-Fe_2_O_3_/NC is calculated in the Table 3, which indicates the better activity of the reaction when magnetic field is applied to a freshly assembled cell.

**Table 3:** Area under the redox peaks of α-Fe_2_O_3_/NC (from Fig. 4c).

Voltage	Peak Area (mA/g.V)	
	Without magnetic field	With magnetic field (fresh cell)
1.6 V	30	57
1.9 V	28.04	47.49
0.8 V	72.3	99

In order to compare the magnetic field influence, cells comprising of commercial α-Fe_2_O_3_ anode are also tested shown in Fig. S12.

**Figure 5:**
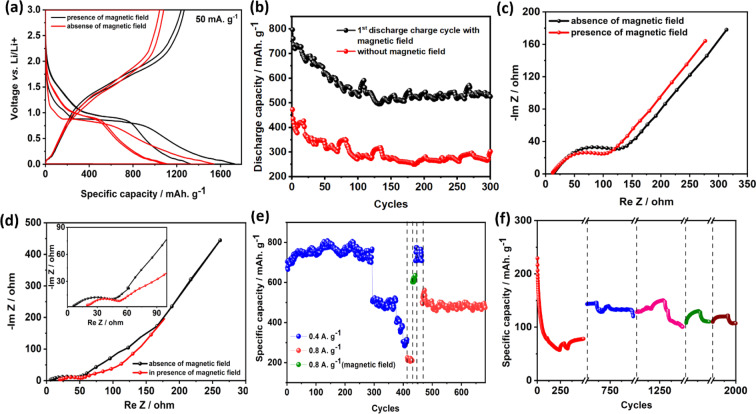
(**a)** Charge discharge profile, **(b)** cyclic stability at 1 A. g^−1^ with application of magnetic field during first discharge-charge cycle and then removed; **(c)** initial impedance, **(d)** impedance after 400 cycles ((inset) magnified plot) in presence and absence of magnetic field **(e)** cyclic stability with magnetic field applied for 10 charge-discharge cycles after 430 cycles and then removed; and **(f)** cyclic stability with magnetic field during single charging at 501^st^, 1001^st^, 1501^st^ and 1751^st^ cycles at 5 A. g^−1^.

Further to investigate the influence of the magnetic field, a new cell was assembled using α-Fe_2_O_3_/NC, and the magnetic field was applied only for the first discharge and charge cycle. Figure 5a,b show the charge-discharge profile and cycling stability of the cell with and without magnetic field at current densities 50 mA. g^−1^ and 1 A. g^−1^. Coulombic efficiency of the 1^st^ cycle enhances from 70% to 73.5% on the application of the magnetic field, indicating lower loss of lithium for SEI formation under the influence of the magnetic field. Specific capacities of 1700 mAh. g^−1^ and 1300 mAh. g^−1^ are achieved at a current density of 50 mA. g^−1^ after 1^st^ and 2^nd^ discharge cycle, whereas the specific capacity of 1500 mAh. g^−1^ and 1100 mAh. g^−1^ are obtained without any magnetic field. This signifies that the application of magnetic field causes about 14% enhancement in the capacity of the magnetic anode (for 1^st^ cycle) and 19% (for 2^nd^ cycle). Even at 1 A. g^−1^, 50% enhancement in the capacity is observed. Kang *et al*. have reported using COMSOL Multiphysics simulations and *in-situ* measurements that in the absence of magnetic field, protrusion on the current collector surface leads to uneven voltage distribution, whereas, with the magnetic field, lithium ion movement is subjected to Lorentz force and hence uniform SEI formation can be achieved leading to capacity enhancement in the LIBs^20^. *Ex-situ* FESEM also shows the porous nature with the cell cycled in presence and absence of magnetic field, Comparative capacity retention after 300 cycles of graphite (Fig. S18) and α-Fe_2_O_3_/NC (with and without magnetic field) are tabulated in Table S2.

Figure 5c,d compare the electrochemical impedance spectra (EIS) for initial cycle and after 400 cycles in the presence and absence of magnetic field respectively. In the absence of the magnetic field, charge transfer resistance increases with cycling due to the slower kinetics of Li^+^ ions across electrode-electrolyte interphase because of the formation of passivating surface layer^28^. Also, diffusion resistance increases due to lesser accessible sites for Li^+^ ions. On the application of the magnetic field, better Li^+^ ion kinetics is realized due to shorter diffusion paths and lower interfacial resistance^20^. In order to understand the influence on the kinetics in detail, dynamic electrochemical impedance spectroscopy (DEIS) measurement is carried out at different charging potentials with at without magnetic field (Fig. S13), which clearly shows the improvement of the kinetics in presence of magnetic field. R_s_ and R_ct_ is calculated in Fig. S13c, which clearly depicts the lower value of R_ct_ and better kinetics owing to the changed slope value. The magnetic field induces convection equivalent to the hydrodynamic effect^29^. In the presence of the magnetic field, both Lorentz and electro-kinetic forces arise due to the interaction of the magnetic and electric field. The magnetic field acts as a tangential electric field created due to the non-equipotential surface of the electrode, whereas electrokinetic stress helps in enhancing the mass transport through the tangential flow from diffused double layer to the bulk solution^29,30^. These forces result in overcoming both convection and diffusion limitation, which can be clearly established from the Fig. 5(c,d). Initially, small changes in the charge transfer resistance and diffusion resistance are observed with and without magnetic field due to the availability of enough diffusion paths. After cycling, diffusion seems to be the limiting factor for the Li^+^ ion transport. Under magnetic field, forced convection helps in overcoming the diffusion limitations^29^. Further, the detailed mechanism for improved mass transport in electrochemical systems due to ferromagnetic particle modified electrode is explained in the supplementary information.

Figure 5e shows cycling of α-Fe_2_O_3_/NC at a current density of 0.4 A. g^−1^ with an initial capacity of 750 mAh. g^−1^ which decreases to 300 mAh. g^−1^ after 400 cycles and further decreases to 200 mAh. g^−1^ in the subsequent 30 cycles at a current density of 0.8 A. g^−1^. At this point, the magnetic field is applied to the anode and operated further only for 10 charge-discharge cycles. The specific capacity is increased to 750 mAh. g^−1^ at 0.4 A. g^−1^, confirming 100% capacity retention. Further cycling for 200 cycles at 0.8 A. g^−1^ results in the constant specific capacity to 500 mAh. g^−1^, which is attributed to the better kinetics of Li^+^ ions near the electrode surface. The *ex-situ* XRD of the cycled and uncycled electrode shows that under the influence of magnetic field with the magnetic α-Fe_2_O_3_/NC anode, intercalation of Li^+^ ions into Fe_2_O_3_ becomes predominant than the transformation from hcp to fcc Fe metal phase which leads to the higher stability of the anode (Fig. S14**)**.

Further to study the influence of the magnetic field during only charging, the magnetic field is applied after 500 cycles at a higher current density of 5 A. g^−1^. On the application of the magnetic field at the anode during 501^st^ charging cycle at 5 A. g^−1^ enhancement of specific capacity from 80 mAh. g^−1^ (36% of its actual capacity) to 150 mAh. g^−1^ which is 68% of its original capacity. Further application of magnetic field at the anode during 1001^st^, 1501^st^ and 1751^st^ charging cycles increases the capacity to 59%, 45% and 45% of actual capacity. The capacity of ~45% can be retained after 2000 cycles (Fig. 5f, Table S3**)**, which can be explained through the hydrodynamic effect created on the application of the magnetic field^29^. On removing the magnetic field after a few cycles, capacity again started to decrease as the electrochemical reactions become diffusion-limited in the absence of forced convection. During charging, in the absence of magnetic field, the saturation magnetization of α-Fe_2_O_3_ decreases with an increase in the coercivity with increasing voltage^21^. But, under the application of magnetic field and in the presence of ferromagnetic anode, magnetic field restrains the demagnetization of the anode^31,32^. Due to the presence of coercivity and remanence magnetization of α-Fe_2_O_3_/NC observed from the VSM measurement (Fig. S2), even after removing the magnetic field, it helps in retaining the spin orientation. Hence for a few cycles, the capacity increases and then decreases when the anode is fully demagnetized. Post-cycling VSM measurement at different charging voltages are performed for the proof-of-concept of the same in presence and absence of magnetic field shown in Figure S15, which also corroborates that even at charging voltage 3 V the electrode indicates in case of ferromagnetic α-Fe_2_O_3_/NC, it cannot be fully demagnetized. Hence, the retention of magnetization is confirmed by applying the magnetic field applied. Further, the magnetic field is applied during discharge cycle after 500 cycles of operation (Fig. S16). During discharge state, Fe_2_O_3_ converts to Fe resulting in high saturation magnetization and low coercivity^21^. Therefore, when the magnetic field is applied only at the 501^st^ discharge cycle, capacity increases from 80 mAh. g^−1^ to 100 mAh. g^−1^ but, after 501^st^ cycle, it started decreasing again in contrast to charging cycle. This is due to the low coercivity of Fe, resulting in easy demagnetization in the absence of the magnetic field. This substantiates that the magnetic properties of anode also play an essential role in LIB performance. This concept is validated for the faded cell as well (Fig. S17).

## Conclusion

In summary, ferromagnetic α-Fe_2_O_3_ nanoparticles coated with N-doped carbon matrix is synthesized employing expired paracetamol drugs as nitrogen rich carbon source by solid state combustion method. Presence of oxygen vacancies results in room temperature ferromagnetism. α-Fe_2_O_3_/NC supports higher intercalation of lithium due to the existence of hcp and fcc phases due to its smaller particle size. This can be enhanced on the application of magnetic field results in higher capacity. The magnetic field applied during the initial charge-discharge cycle for new cell can almost give 30% and 50% enhancement after 1^st^ and 100^th^ discharge cycles. For a cycled cell, single charging with the magnetic field can bring back its capacity up to 45% even after 2000 cycles. This is due to the field-controlled enhancement of convection and diffusion in presence of magnetic nanoparticles modified electrode, which help in better Li^+^ ion transport and reduces the electrochemical overpotentials in the presence of the Lorentz and electrokinetic force leads to magnetic field gradient associated with it. This study substantiates that for both fresh and cycled cell, cycle life and capacity of the battery can be improved with the magnetic α-Fe_2_O_3_/NC anode and applied magnetic field. This concept can solve the problems of capacity fading and expensive process of recycling of LIBs. Moreover, utilization of the expired drugs used for the synthesis of efficient anode material can solve the problem of environmental pollution caused by their disposal.

## Methods

### Chemicals used

Iron nitrate nonahydrate (Fe(NO_3_)_3_.9H_2_O), Sodium hydroxymethyl cellulose (Na-CMC) and commercial α-Fe_2_O_3_ powder were purchased from Merck. Conducting graphite, Lithium metal are obtained from Alfa-aesar. Paracetamol (500 mg, acetaminophen) manufactured by Glaxo SmithKline Pharmaceuticals Ltd. was used in this study after its expiry. All chemicals were used as received without further purification.

### Synthesis of N-doped carbon coated α-Fe_2_O_3_ nanoparticles (α-Fe_2_O_3_/NC)

**Scheme 1:**
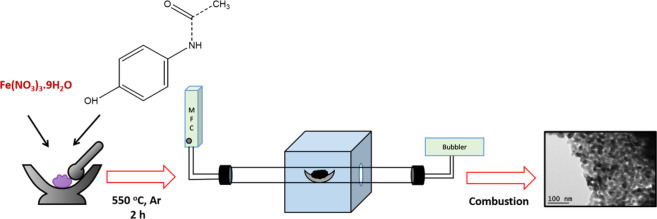
Synthesis of α-Fe_2_O_3_/NC.

α-Fe_2_O_3_/NC was prepared by a simple solid-state combustion method in the presence of expired paracetamol drugs. 750 mg of iron nitrate salt was mixed with 500 mg of expired paracetamol drug and loaded into alumina boat at the centre of the tube furnace. The temperature of the furnace is raised to 550 °C and maintained for 2 h under the flow of argon. The furnace was then slowly cooled to 300 °C and the sample was exposed to atmosphere to initiate combustion reaction (Scheme 1).

### Electrochemical characterization

The slurry electrode was prepared by mixing α-Fe_2_O_3_/NC with conducting graphite and binder in 75:10:15 ratios. Binders used for the slurry preparation were sodium carboxymethyl cellulose (Na-CMC) and water. Working electrodes were prepared by coating the slurry on copper foil and further dried at 80 °C overnight in a vacuum oven. Dried electrodes then cut into circular disks (diameter of 12 mm). Active material loading was maintained to 0.75–1 mg cm^−2^. 2032-Coin cells were assembled in an argon-filled glovebox, with maintained moisture and O_2_ levels below 0.1 ppm. Lithium metal and glass fibre membrane (Whatman GF/C) were used as the reference electrode and the separator, respectively. Commercial 1 M LiPF_6_ dissolved in ethylene carbonate/dimethyl carbonate (EC/DMC 1:1 v/v) was used as the electrolyte. Assembled cells were cycled at various current densities (0.05–20 A. g^−1^) between 0.005 and 3 V using Bio-Logic BCS 810 battery cycler. Potentiostatic electrochemical impedance spectroscopy (PEIS) was conducted by applying an AC signal of amplitude 5 mV in the frequency range of 10 mHz -10 kHz. Dynamic electrochemical impedance spectroscopy (DEIS) was conducted by applying an AC signal of amplitude 5 mV in the frequency range of 1 MHz -1 Hz. Cyclic voltammetry was carried out in the potential range of 0.005–3 V at different scan rates of 0.1–0.8 mV. s^−1^. The external magnetic field of 0.15 T was applied at the anode on freshly assembled as well as cycled and faded cells while charging/charging-discharging.

### Physical characterization

X-ray diffraction (XRD) study for structural and phase analysis was carried out by Rigaku diffractometer Cu-Kα radiation source. Morphological studies and elemental mapping were carried out using Inspect F scanning electron microscopy (FESEM) (30 kV). HRTEM, STEM-HAADF analysis were recorded using JEOL scanning transmission electron microscope. Room temperature magnetization was measured within −7 T to +7 T in quantum design MPMS SQUID VSM from −7T to 7 T. X-ray photoelectron spectroscopy (XPS) was done using SPECSLAB instrument and PHOIBOS 100MCD analyzer with polychromatic Mg Kα X-rays (hν = 1253.6 eV) operated at ultrahigh vacuum (10^−9^ mbar). Thermogravimetric analysis (TGA) was performed using TA instruments in an air atmosphere at 20 °C. min^−1^ heating rate.

### *Ex-situ* post-mortem analysis

To characterize structural change after different discharge cycles and discharge voltages, cycled cells were disassembled inside Ar-filled glovebox. Electrodes were then washed with ethylene carbonate solvent to remove lithium salt in the residual electrolyte and dried thoroughly inside the glove box. Dried electrodes were then cut and used for *ex-situ* FESEM and *ex-situ* XRD analysis.

### Supporting Information

Supporting Information shows details of precursor characterization, sample preparation and the data analysis.

## Supplementary information

Supplementary Information.
