# Supplementary material for: Magnetic field assisted high capacity durable Li-ion battery using magnetic α-Fe2O3 nanoparticles decorated expired drug derived N-doped carbon anode
Source: Sci Rep. 2020 Jun 19;10:9945. doi: 10.1038/s41598-020-67042-1 (PMC7305126; doi:10.1038/s41598-020-67042-1)
Supplement: Supplementary file 1 — Supplementary Information. [file 41598_2020_67042_MOESM1_ESM.doc]

Supplementary Information

**Magnetic field assisted high capacity durable Li-ion battery using magnetic α-Fe_2_O_3_ nanoparticles decorated expired drug derived N-doped carbon anode**

*Dipsikha Ganguly, Ajay Piriya V.S, Anamika Ghosh and Sundara Ramaprabhu^*^*

Alternative Energy and Nanotechnology Laboratory, Department of Physics,

India Institute of Technology Madras, Chennai- 600036, India

**X-ray photoelectron spectroscopy (XPS)**

XPS spectra of α-Fe_2_O_3_/NC and commercial α-Fe_2_O_3_ are shown in **figure S1**. The deconvoluted C 1s spectrum shows the presence of sp^3^ carbon, C=O, along with C-N species in the α-Fe_2_O_3_/NC sample. Successful doping of nitrogen is confirmed from the N 1s spectra of the synthesized sample. Deconvoluted N 1s spectrum shows four types of nitrogen moieties; pyrrolic N (400.3 eV) to be the highest, followed by graphitic (401.8 eV), pyridinic N (398.3 eV) and oxidized N (405.1 eV)^1^. O 1s spectrum indicates the existence of Fe-O-C bond, C-O and N-O bond, and Fe-O bond in the α-Fe_2_O_3_/NC sample, whereas for O 1s spectrum of pure Fe_2_O_3_, only Fe-O bond can be seen from the **figure S1b and S1e**. Deconvoluted Fe 2p spectra of both synthesized α-Fe_2_O_3_/NC and commercial α-Fe_2_O_3_ are shown in **figure S1c and S1f**. Fe 2p_3/2_ and Fe2p_1/2_  obtained at 724.9 eV and 711.3 eV for α-Fe_2_O_3_/NC sample corresponds to Fe^3+^ states^2,3^. For commercial α-Fe_2_O_3_, Fe 2p_3/2_ and Fe2p_1/2_  peaks are observed at 721.7 eV and 708.9 eV. The shift of Fe2p_3/2_ and Fe2p_1/2_ peaks in α-Fe_2_O_3_/NC in comparison with α-Fe_2_O_3_ indicates the presence of oxygen vacancies in the sample, which corroborates well with the XRD investigation^3^. The separation of Fe2p_3/2_ and Fe2p_1/2_ peaks in α-Fe_2_O_3_/NC is around 13.6 eV. N and C ratio is estimated from the XPS data is found to be 0.2 at.%.


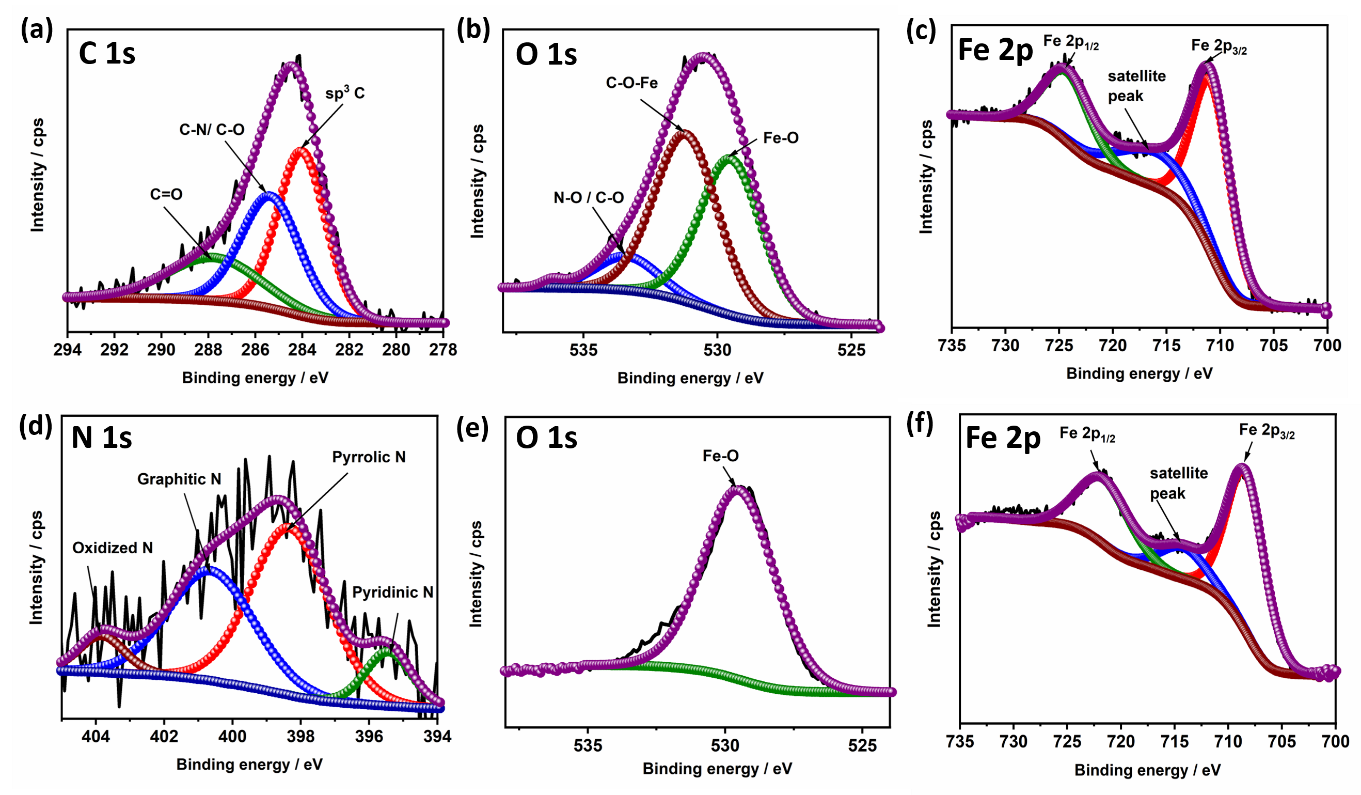


**Figure S1.** Deconvoluted XPS spectra of (a) C 1s (b) O 1s (c) Fe 2p (d) N 1s of α-Fe_2_O_3_/NC; Deconvoluted XPS spectra of (e) O 1s (f) Fe 2p of pure α-Fe_2_O_3_.

**TGA analysis**

Thermogravimetric analysis from room temperature to 800 °C in air atmosphere was performed for α-Fe_2_O_3_/NC sample. Initial weight loss of 4 wt.% observed 100 °C of α-Fe_2_O_3_/NC corresponds desorption of moisture present in the sample. Significant mass loss started at ~300 °C to 500 °C, corresponds to carbon and nitrogen decomposition in the presence of oxygen. After 500 °C, no significant change is observed in the sample, and the residual weight of the α-Fe_2_O_3_ is estimated to be 70 wt.% (**figure S2**).

**
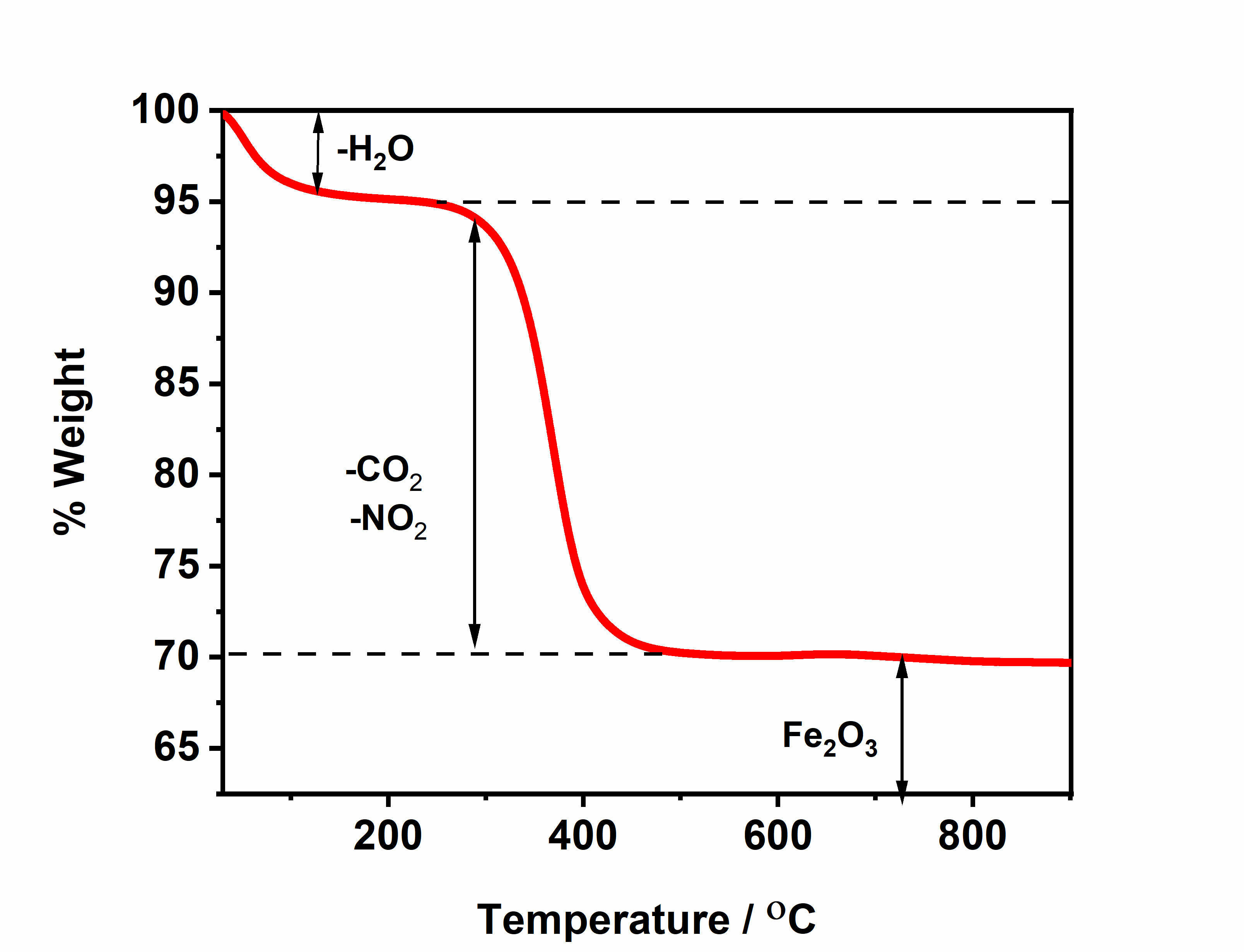
**

**Figure S2.** TGA curve of α-Fe_2_O_3_/NC

**Raman Data**

Raman data of α-Fe_2_O_3_/NC sample is shown in **figure S3**. D band and G bands are centred at 1340 cm^-1^ and 1576 cm^-1^. I_D_/I_G_ ratio is calculated to be ~1.97 from the area of the deconvoluted peaks, which also signifies the disorders of the carbon present in the sample. Peaks at 215 and 475 cm^-1^ correspond to the A_1g_ mode, whereas peaks at 278, 391, 601, and 658 cm^-1^ attributes to the E_g_ mode of hematite structure^4^.


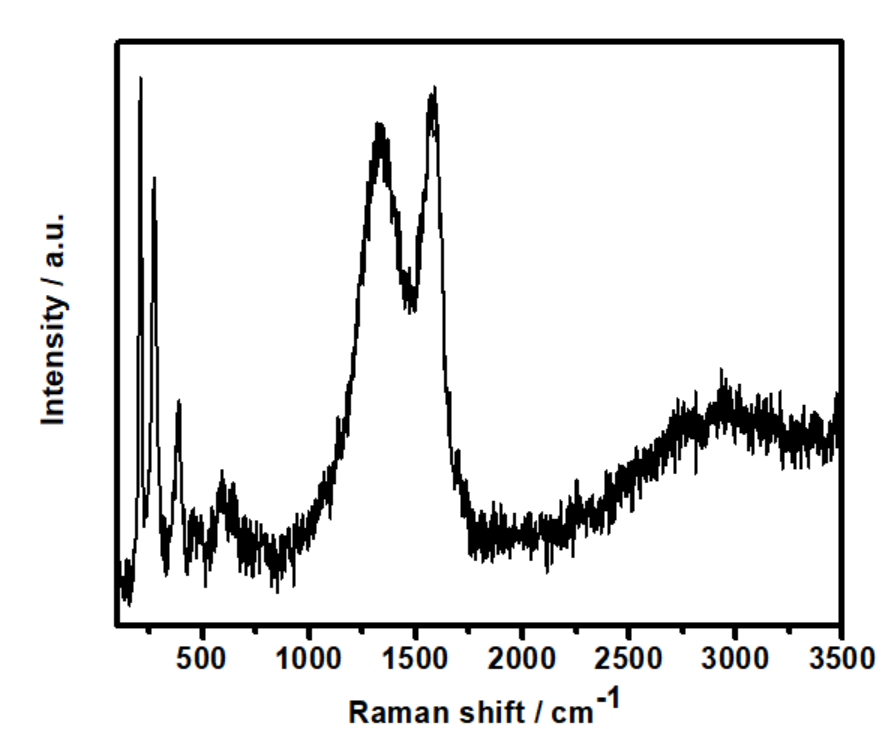


**Figure S3.** Raman data of α-Fe_2_O_3_/NC

**Magnetic measurement study**

M *vs.* H plot obtained at 300 K shows the saturation magnetization (M_s_) of 16 emu. g^-1^ for α-Fe_2_O_3_/NC (**figure S4**). In pristine α-Fe_2_O_3_, about specific c planes, Fe^3+^ ions are ferromagnetically coupled, and in between the planes are antiferromagnetically coupled^5^. The presence of oxygen vacancies introduced in the α-Fe_2_O_3_/NC, sample led to the increase of the magnetocrystalline anisotropy of the material due to the broken Fe^3+^-O-Fe^3+^ superexchange bonds. Also, interparticle interaction between surface and neighbouring atoms influences the exchange anisotropy due to the reduced particle size^6^. As a result, magnetism has been induced in α-Fe_2_O_3_/NC. Remanence magnetization indicates the presence of ferrimagnetic phase along with a strong ferromagnetic phase with a coercivity of ~115 Oe.


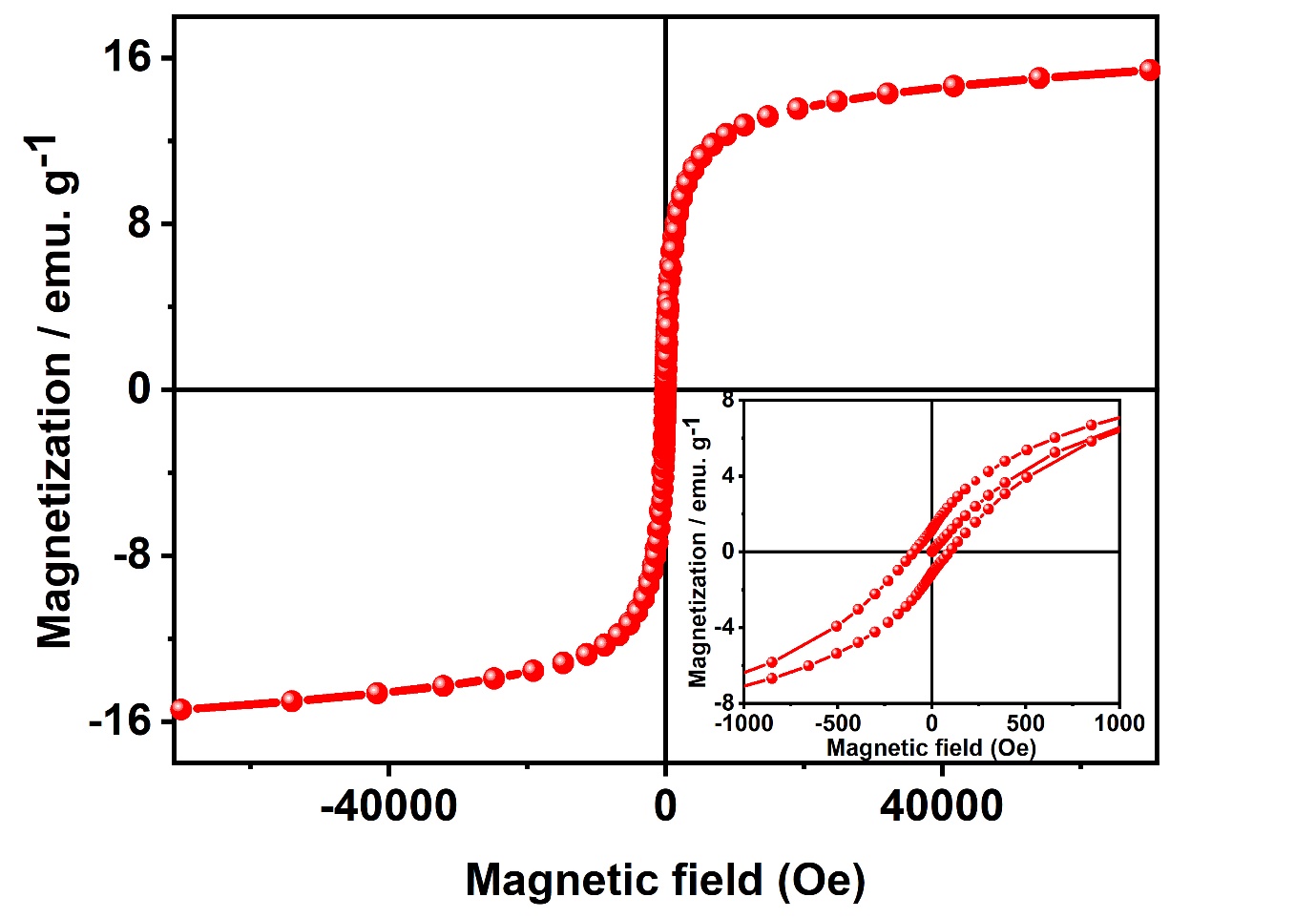


**Figure S4.** M *vs*. H plot of α-Fe_2_O_3_/NC at room temperature

**FESEM Analysis**

FESEM micrograph (**figure S5**) of α-Fe_2_O_3_/NC confirms the highly porous nature of the synthesised material. High magnification image shows ultrasmall nanoparticles (10-20 nm) distributed over N doped sheet-like carbon structure, which can be easily be seen from the HRTEM image in **figure 2**.


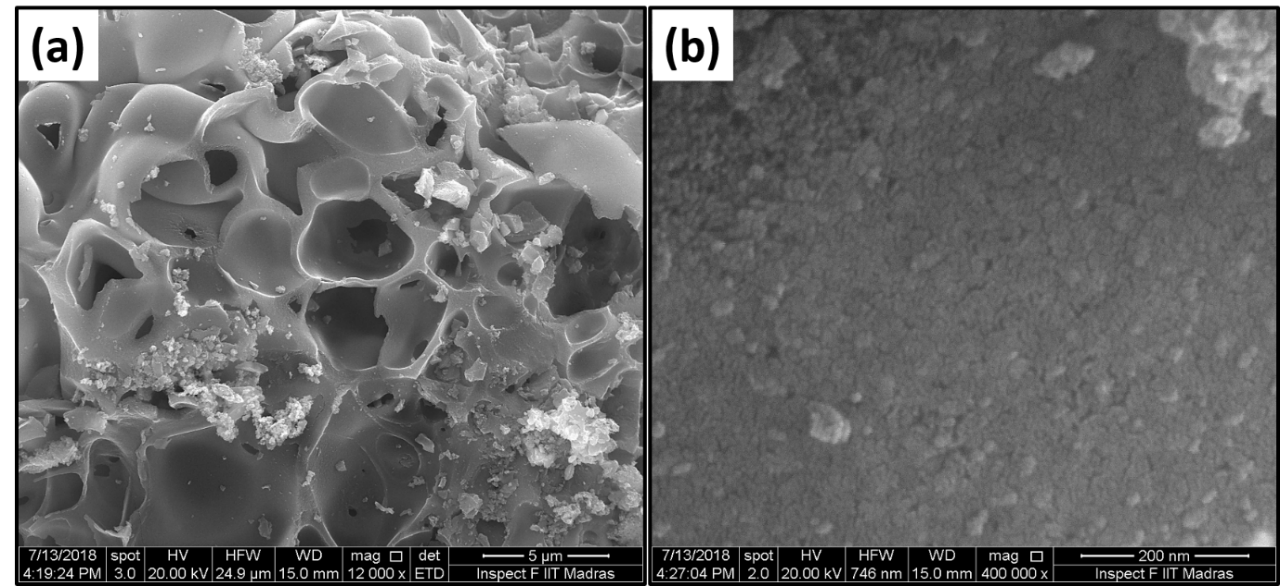


**Figure S5.** FESEM image of α-Fe_2_O_3_/NC (a) low magnification (b) high magnification

**BET analysis**

From BET adsorption-desorption isotherm shown in **figure S6,** it is obvious that α-Fe_2_O_3_/NC shows type IV N_2_ adsorption-desorption isotherm, which implies the presence of micropores (< 2 nm) in the structure^7^. For high pressure region (P/Po > 0.5) hysteresis loop with noticeable edges signifies the formation of mesopores, whereas the low-pressure region the knee with edges confirms the formation of micropores. Pore size distribution (PSD) calculated using BJH model illustrates the presence of micropores in the sample. The surface area for the sample is found to be 179 m^2^. g^-1^, which is high for metal oxides. Also, the average pore size from BJH (Barrett, Joyner, and Halenda) method and non-linear density functional theory (DFT) is found to be 2- 3 nm.


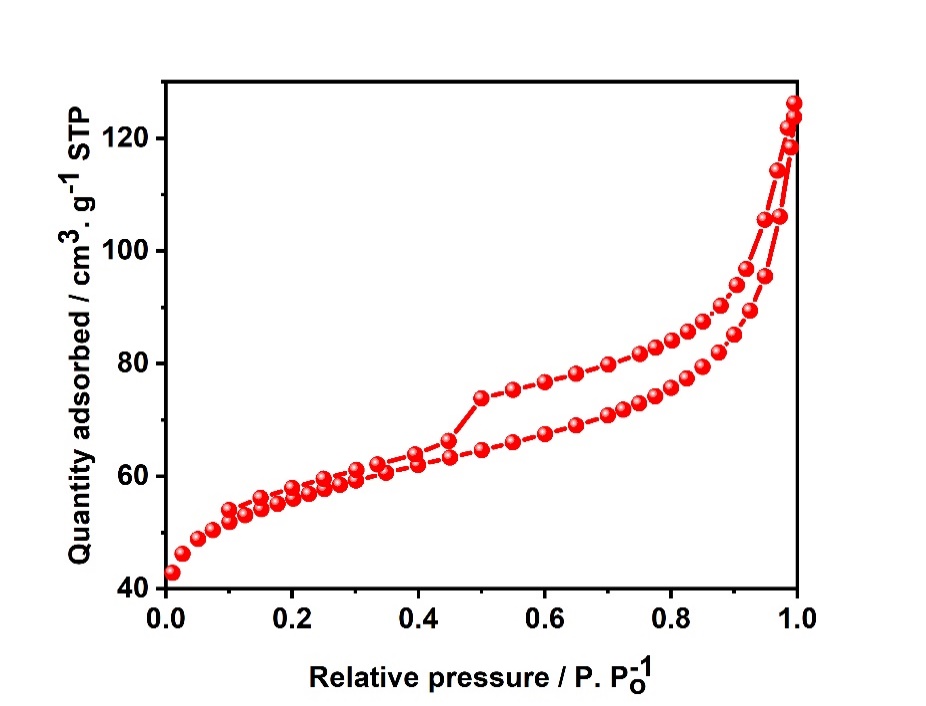


**Figure S6.** BET isotherm of α-Fe_2_O_3_/NC

**
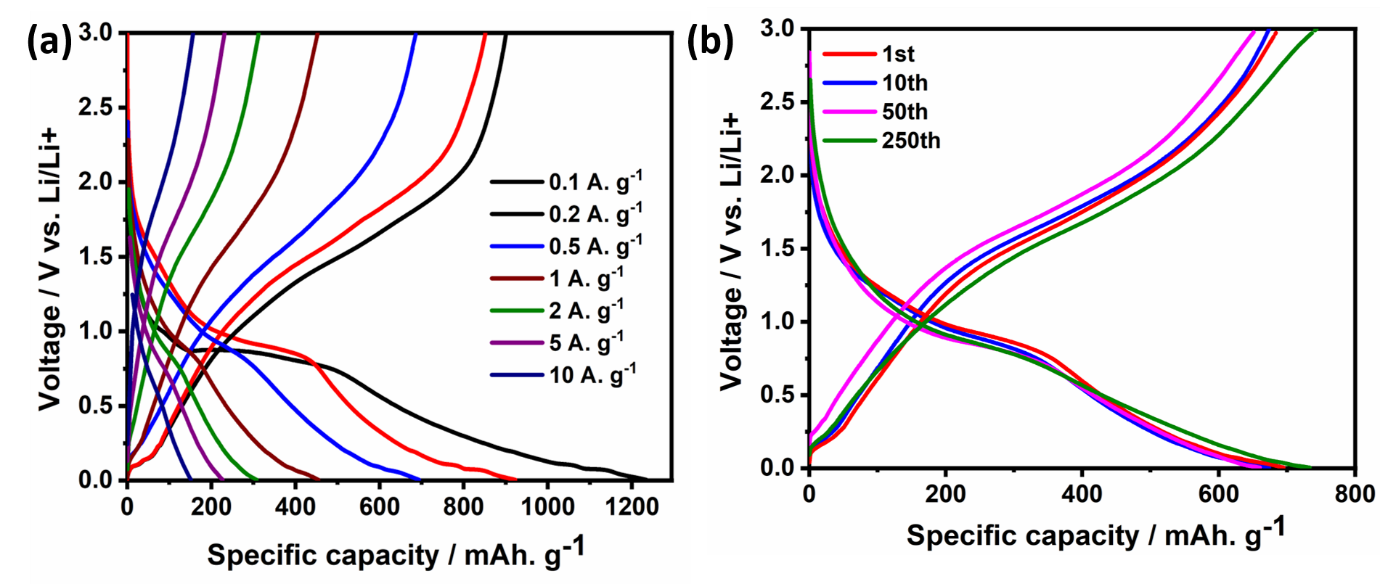
**

**Figure S7**. Charge discharge profiles **(a)** at different current densities (0.1-10 A. g^-1^) **(b)** at a current density of 0.4 A. g^-1^

**Figure S7a** shows the first charge-discharge cycles obtained at different current densities (0.1-10 A. g^-1^). Specific capacities of 1200 mAh. g^-1^, 900 mAh. g^-1^, 650 mAh. g^-1^, 450 mAh. g^-1^, 300 mAh. g^-1^, 220 mAh. g^-1^, and 180 mAh. g^-1^ are obtained at current densities of 0.1 A. g^-1^, 0.2 A. g^-1^, 0.5 A. g^-1^, 1 A. g^-1^, 2 A. g^-1^, 5 A. g^-1^, and 10 A. g^-1^ respectively. The first discharge cycle at 0.1 A. g^-1^ indicates the SEI layer formation due to the decomposition of the electrolyte. **Figure S7 b** shows charge-discharge profiles after 1^st^, 10^th^, 50^th^ and 250^th^ cycles at a current density of 0.4 A. g^-1^. Before operating at 0.4 A. g^-1^ the cell was cycled for 2 charge-discharge cycles at a low current density of 0.05 A. g^-^1 for stable SEI formation. Retention of ~100% capacity even after 250 cycles indicate the good stability of the anode material.

**Ex-situ FESEM analysis of cycled cells**

FESEM analyses of the cycled cells discharged at three different voltages (0.005 V, 0.5 V and 1.6 V) along with the uncycled cell have been shown in **figure S8**. Structural changes from the uncycled and cycled electrodes observed from FESEM give a better understanding of the step by step formation of the SEI layer.


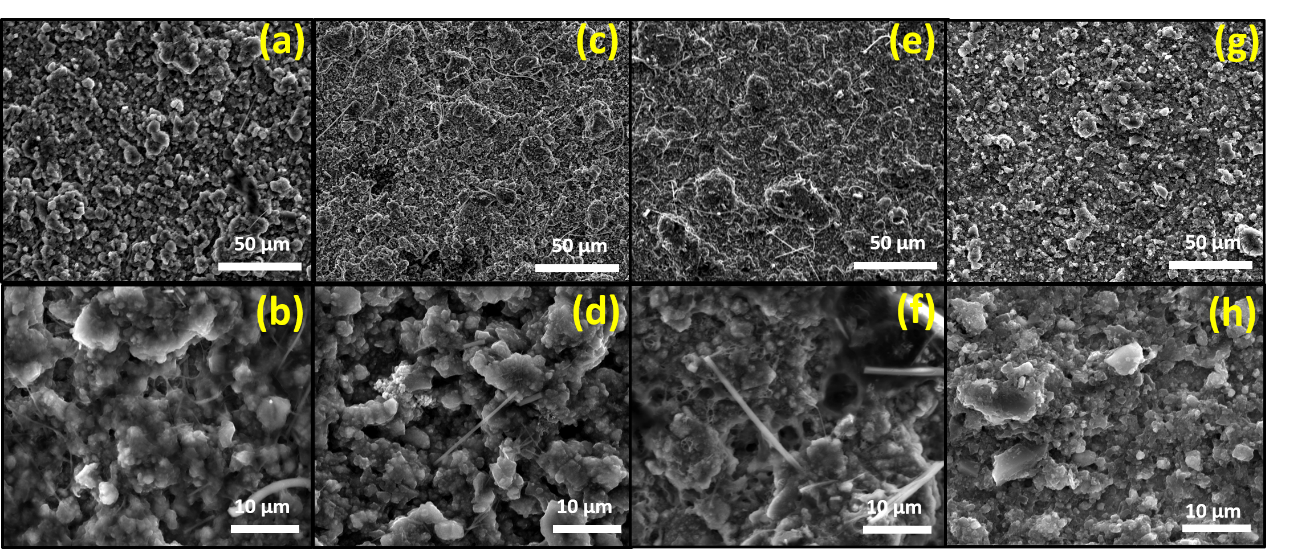


**Figure S8**. FESEM images of cycled cell discharged from 3 V to **(a-b)** 0.005 V **(c-d)** 0.5 V **(e-f)** 1.6 V; **(g-h)** uncycled electrode.

FESEM images of the cycled cells after 3 discharge cycles in the presence and in the absence of magnetic field are shown in **Figure S9**. In presence of magnetic field, porous nature is observed in the cycled electrode. Further, elemental mapping is carried out to understand the phase formation after 3 cycles in presence and absence of magnetic field.


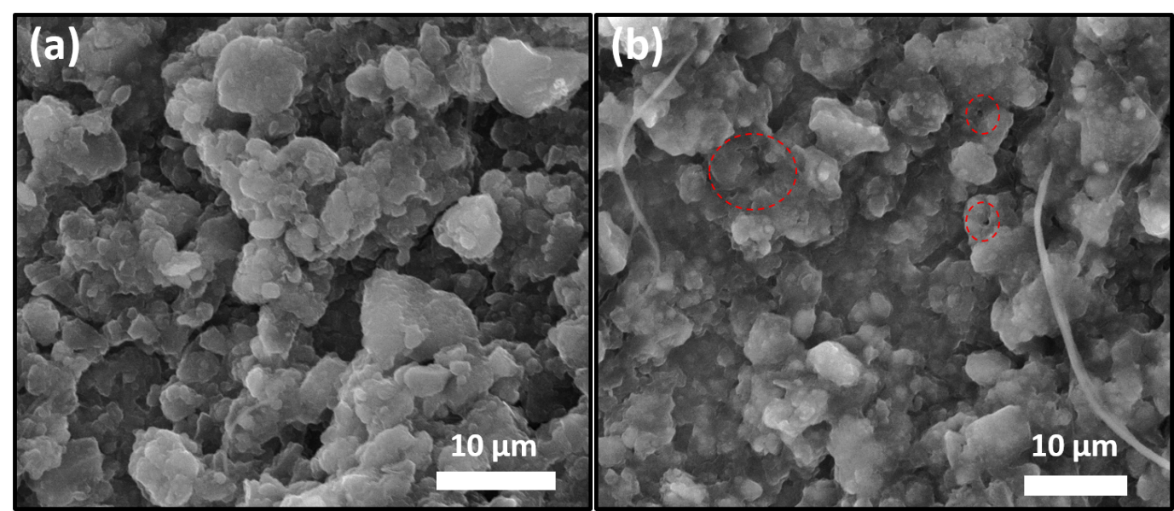


**Figure S9**. FESEM images of cycled cell discharged from 3 V to 0.005 V (a) in absence magnetic field (b) in presence of magnetic field (red dotted lines : pores in the structure)

Elemental mapping for the same has been shown in **figure S10** and **S11**. **Figure S10 a** and **11 b** show “elements to phase” plot of the cycled electrodes, which is the reflection of the number of different phases present and the uniformity of distribution of the elements. In the absence of magnetic field 7 phases can be seen highlighted in different colours, whereas with magnetic field it has reduced down to two only. It substantiates the uniform distribution of elements in the presence of magnetic field and the uniformity of the SEI layer. **Figure S10 c** and **Figure S11 c** shows the overlay image depicting the overall distribution of all the elements over this selected area.


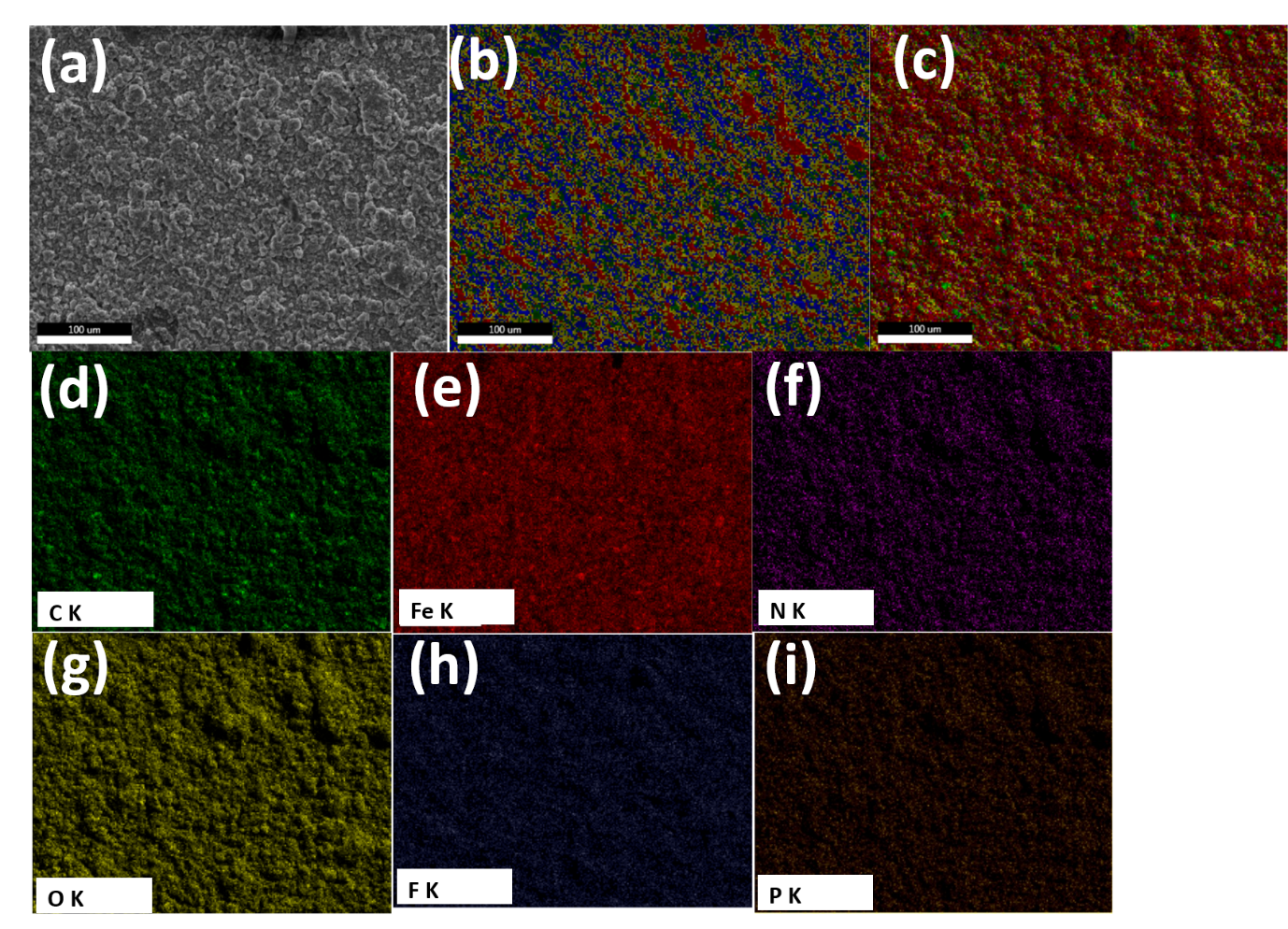


**Figure S10** Elemental mapping of cycled cell in the absence of magnetic field (a) selected area (b) element to phase mapping (c) elemental mapping (d) C K (e) Fe K (f) N K (g) O K (h) F K (i) P K


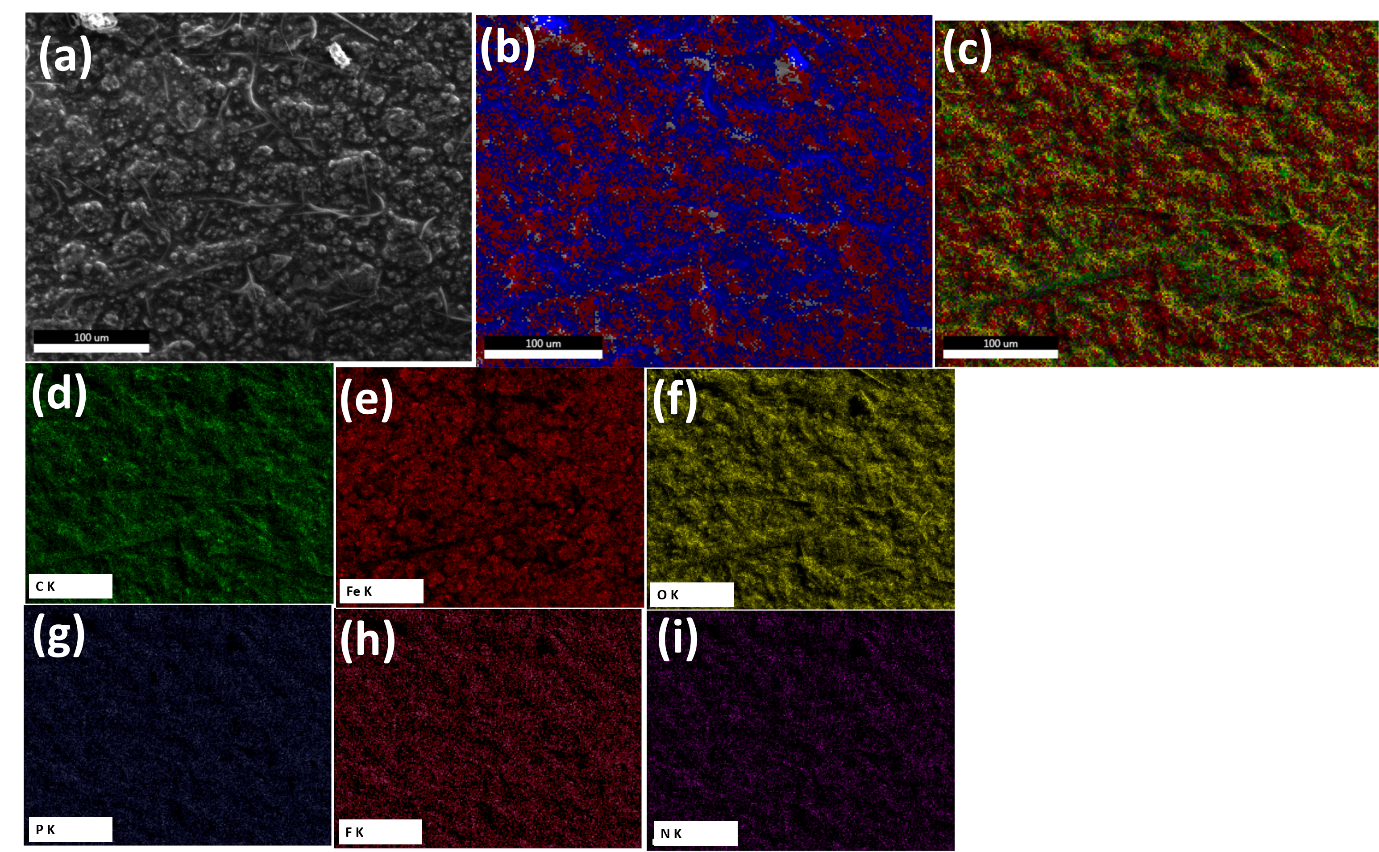


**Figure S11** Elemental mapping of cycled cell in the presence of magnetic field (a) selected area (b) element to phase mapping (c) elemental mapping (d) C K (e) Fe K (f) O K (g) P K (h) F K (i) N K

**CV study of commercial α-Fe_2_O_3_**

CV study of commercial α-Fe_2_O_3_ shown in **figure S12** reveals that with the application of magnetic field, discharge profile of α-Fe_2_O_3_ shows prominent influence compared to charge profile. During discharging, α-Fe_2_O_3_ gets converted to Fe, which is ferromagnetic in nature, whereas during charging it is again getting converted to α-Fe_2_O_3_ which is antiferromagnetic. So, no noticeable change is observed in case of oxidation peaks with this low magnetic field, which is expected. Scan rate variation of α-Fe_2_O_3_ is also shown in **figure S12 c,** shows increased current oxidation peaks, whereas the increase in the reduction peak with scan rate is not too high confirming increase in the diffusion resistance during reduction of Fe^3+^ to Fe^0^ phase. Prominent difference in the peaks are observed when compared to α-Fe_2_O_3_/NC.


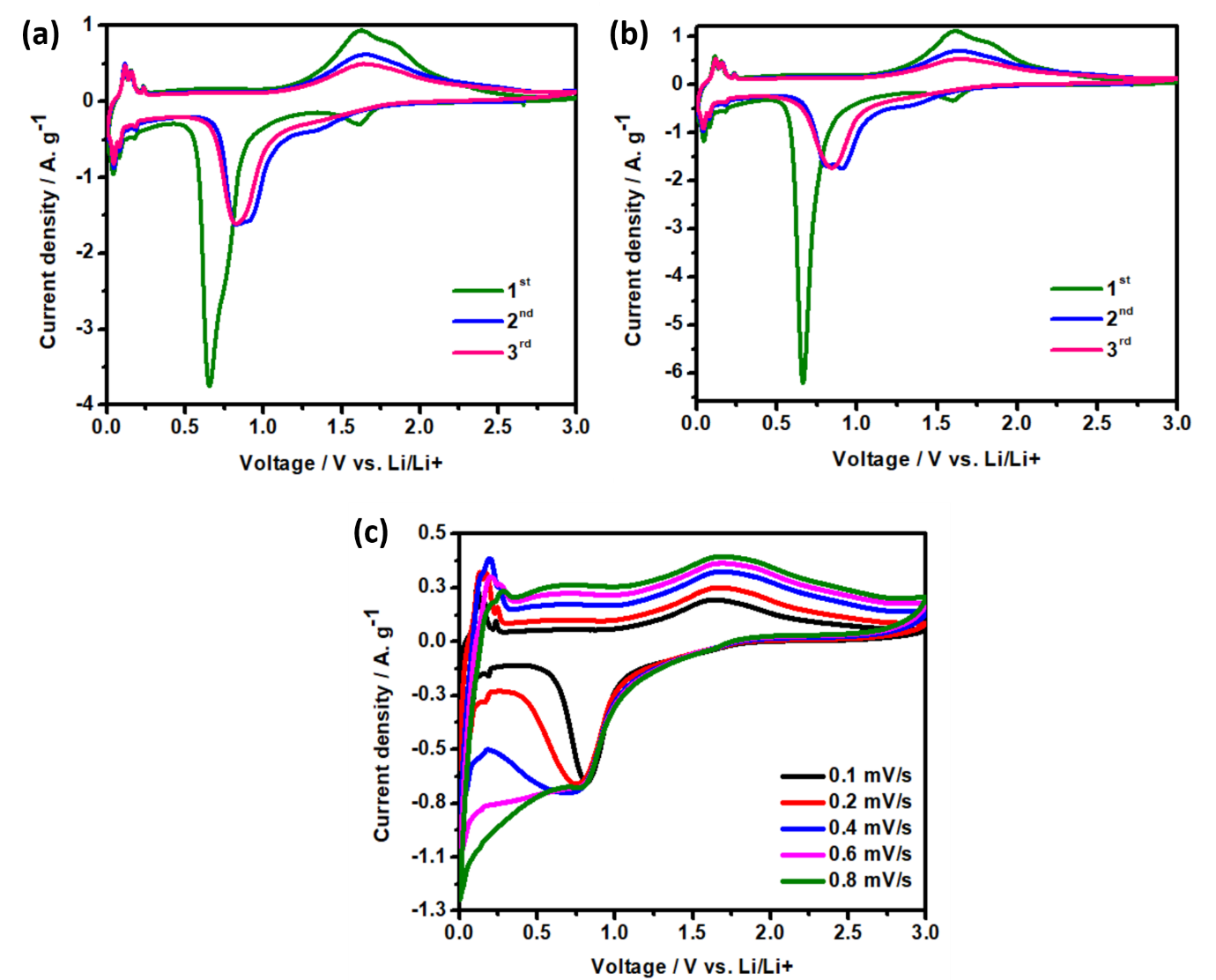


**Figure S12.** CV of commercial α-Fe_2_O_3_ at a scan rate of 0.1 mV/s (a) without magnetic field (b) with magnetic field (c) scan rate variation without magnetic field.

**DEIS plot of α-Fe_2_O_3_/NC**


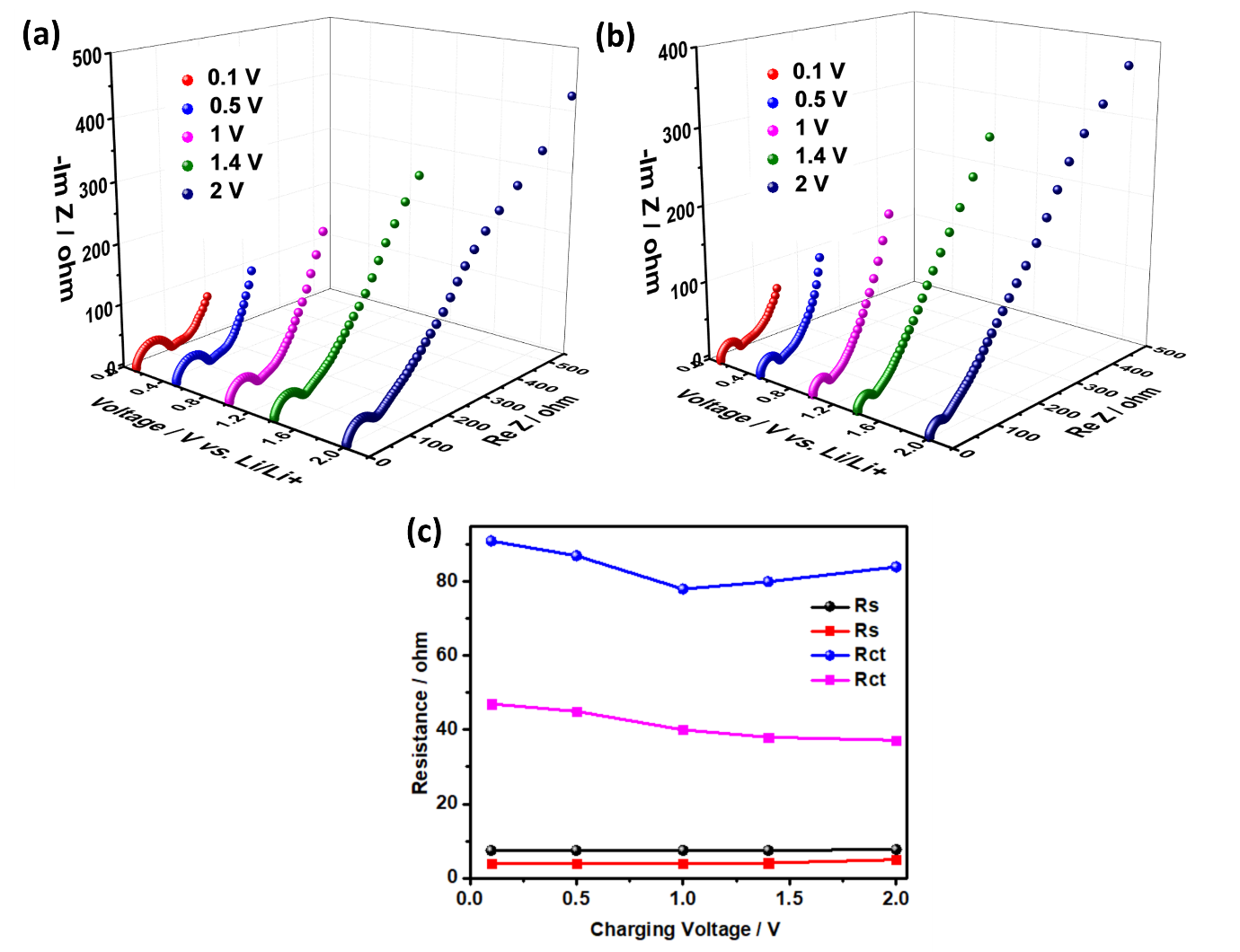


**Figure S13**. DEIS plot of α-Fe_2_O_3_/NC for charging at different voltage (a) without magnetic field (b) with magnetic field (c) R_s_ and R_ct_ parameters for DEIS plot (●) without magnetic field (■) with magnetic field

**Ex-situ XRD investigation of the cycled cells**

Detailed ex-situ XRD studies are carried out to probe the influence of the magnetic field on the LIB using Fe_2_O_3_/NC anode. **Figure S14a** shows XRD of the uncycled fresh cells after coating. **Figure S14b** and **S14c** show XRD patterns of the cycled cell after three discharge cycles from OCV to 0.005 V in the presence and absence of magnetic field, respectively. Peaks for Li_2_CO_3_ arise due to SEI formation. Lower angle shift in the peaks corresponds to α-Fe_2_O_3_ phase confirms the stress-induced lattice expansion because of Li^+^ ion intercalation into the structure^8^. In the absence of magnetic field, metallic Fe peaks are sharper and narrower, indicating the increase in the crystallite size with cycling. In contrast, in the presence of the magnetic field, broader metallic Fe peaks are observed which proves that the magnetic field inhibits the increase in the crystallite size. In addition, with increasing cycles, the average peak intensity ratio of Fe and α-Fe_2_O_3_ phase signifies that in the absence of magnetic field, the conversion reaction of Fe^3+^ to Fe^0^ is faster compared to the intercalation, whereas in the applied magnetic field, intercalation of Li^+^ ions into α-Fe_2_O_3_ is the governing mechanism for LIB. This also corroborates well with the cyclic voltammogram and charge-discharge studies already explained.


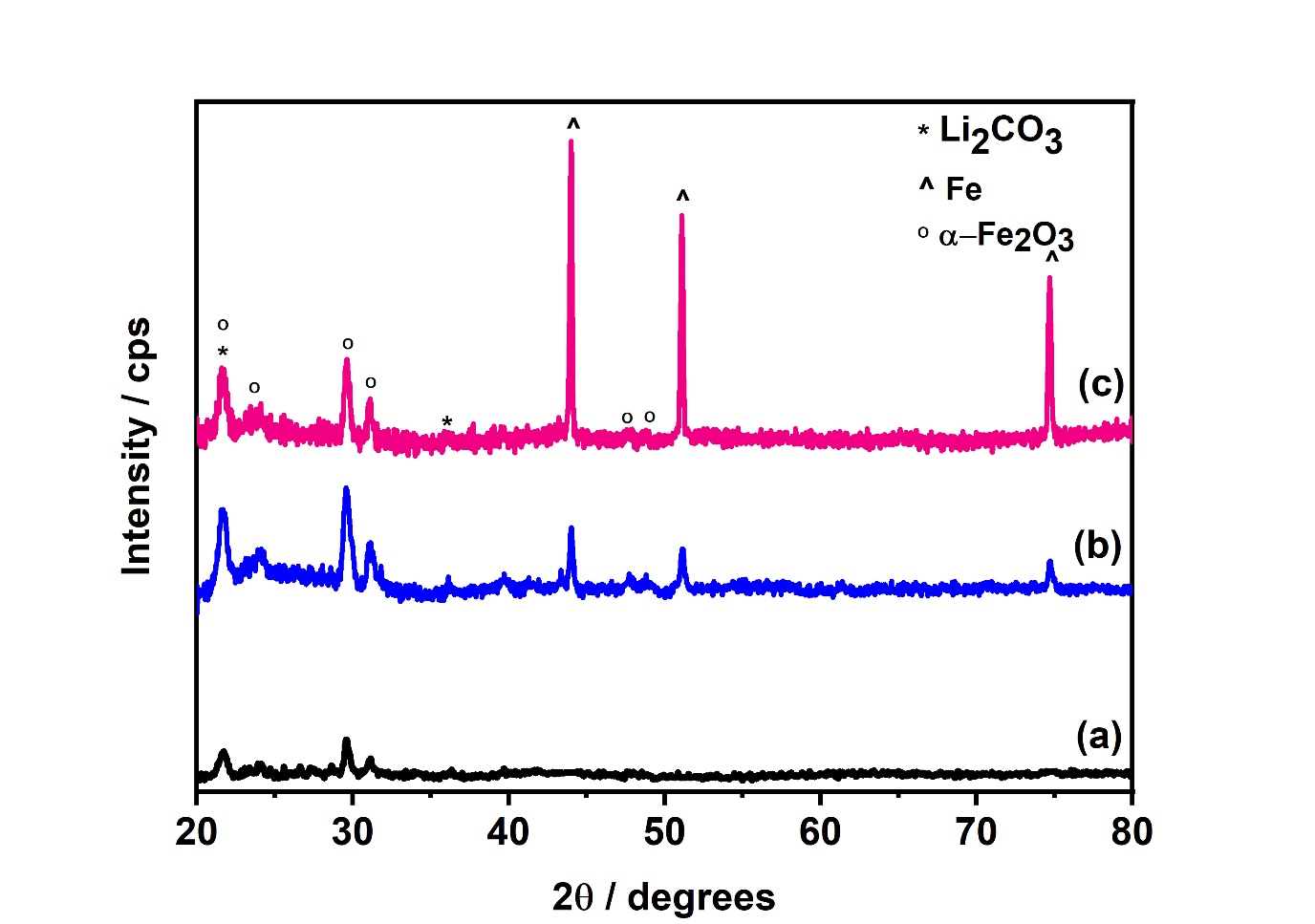


**Figure S14.** XRD of (a) freshly coated electrode (b) cycled cell after 3^rd^ discharge at 0.005 V in the presence of magnetic field (c) cycled cell after 3^rd^ discharge at 0.005 V in the absence of magnetic field

**Mechanism for magnetohydrodynamic effect for improved mass transport for ferromagnetic electrode material:**

Compton et al^9^ has shown the influence of magnetic nanoparticles in accelerating the transport of the reactant and products at electrode vicinity through experimental and theoretical simulations, which might also be useful here to explain the phenomenon observed.

Basically, Magnetohydrodynamic effect is explained by Lorentz force (f_L_)^10^ , where

f_L_ = j x B. (j = current density and B = magnetic field)

For an electrochemical system, magnetic susceptibility of the electrolyte solution (χ_sol_) changes drastically near the electrode due to the concentration gradients of paramagnetic species arise due to the transfer of the paramagnetic species across the electrode through electrolyte. This gives rise to another significant force i.e. magnetic field gradient force (f_m_) that varies across the diffusion layer. Depending on the high magnetic field gradients present in that region, convective flow may be induced near the electrode. In case of electrochemical systems, the influence of f_m_ on the convective mass transport can be described by the following equation^11^

∇× f_m_ = (χ_sol_ / 2μ_o_)/δ. (∇c_para_ x (∇B^2^))

where, μ_o_ is permeability, ∇c_para_= the concentration change in the diffusion layer of thickness δ.

In presence of the electrode modified with magnetic materials, large magnetic gradient force can be created in addition to the Lorentz force. Due to the magnetization of Fe_3_O_4_, enhanced mass transfer is obtained in the electrochemical systems which is also validated with Comsol Multiphysics simulation, which showed magnetic field gradient close to the vicinity of magnetic nanoparticles is higher and drops drastically with increasing distance.


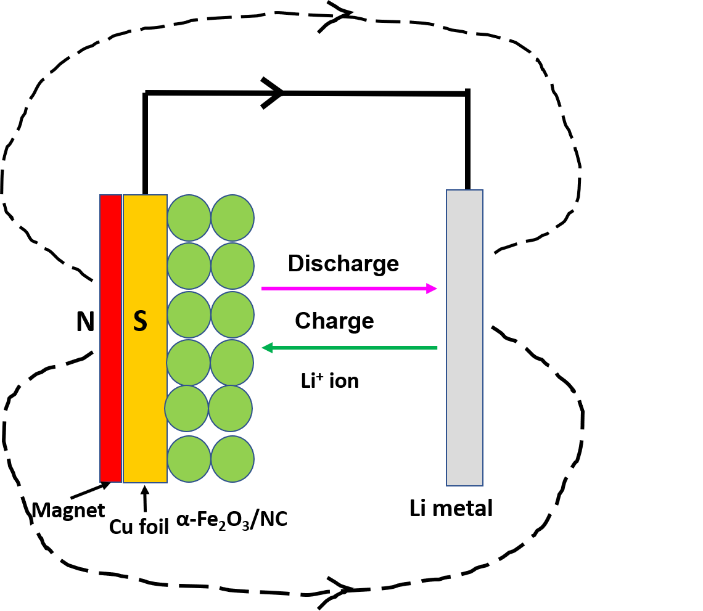


**Scheme S1.** Lithium ion battery with ferromagnetic electrode, magnetic field and Li^+^ ion as paramagnetic species

Here, the same concept is also applicable for ferromagnetic α-Fe_2_O_3_/NC systems where paramagnetic species are Li^+^ ion (scheme S1). In presence of magnetic α-Fe_2_O_3_/NC, magnetic field gradient will also increase owing to the magnetization of α-Fe_2_O_3_/NC, which results in the enhanced mass transport in the electrochemical systems. In addition, this depends on the diffusion layer thickness. With simulations, it has been shown the magnetic field gradient decreases far from the magnetic nanoparticles, which is the reason that for cycled electrode the influence found to be less prominent compared to the electrode where the magnetic field is applied at the first cycle (**figure 4 b and c**)

**Post-cycling VSM study:**

Post cycling magnetization studies at different charge voltages are performed in order to have a proof-of-concept of the magnetic switching described. It can be seen that in presence of the magnetic field, magnetization decreases and coercivity increases as expected from the magnetic switching^8^. But, due to the ferromagnetic nature of the anode, magnetization of the electrodes do not become close to 0 even at 3 V. For the post magnetization study, assembled cells are first discharged at 0.005 V and then charged at different voltages (1.6 V and 3 V) and further de-assembled to record the VSM data. Cell charged at 1.6 V shows the magnetization of ~4.5 emu. g^-1^ with a coercivity of 289 Oe. Cell charged at 3 V also shows ferromagnetic behaviour with coercivity of 500 Oe and magnetization of active material calculated to be ~1.8 emu. g^-1^ (**figure S15 a**). Magnetization study for the cells without magnetic field at charging voltage of 1.6 V is also performed and observed the magnetization and coercivity to be 2 emu. g^-1^ and 500 Oe respectively (**figure S15 b**), which clearly show that the magnetic field influences the intrinsic magnetization property of the electrode, thus helps in achieving better performance of the battery discussed earlier.


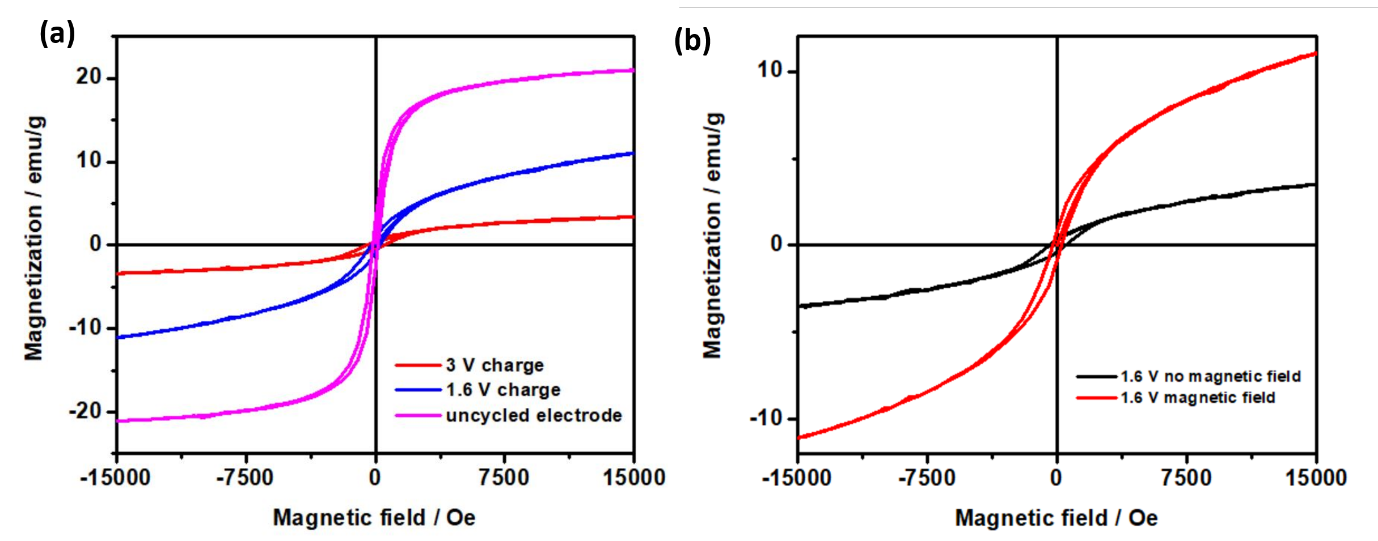


**Figure S15.** Post VSM analysis of the cycled electrodes (a) (under magnetic field) at charged state at 1.6 V and 3 V (b) comparison at 1.6 V charge state with and without magnetic field.

**Magnetic field applied during single discharge**

**Figure S16** shows the increase in the specific capacity during application of magnetic field at 501^st^ discharge cycle. Capacity enhancement corresponds to lower overpotential during Li^+^ ions interaction, which can be due to the reduced diffusion resistance. But, unlike charging, on removing magnetic field capacity started decreasing immediately in the consequent cycles, which confirms the coercivity plays a significant role during the effect of magnetic field and demagnetization. During discharge, Fe conversion takes place at the anode, which is highly ferromagnetic but with a very low coercivity^8^. Lower coercivity corresponds to no memory effect. So, on removing the magnetic field, it immediately demagnetizes, and capacity decreases as usual manner.

**
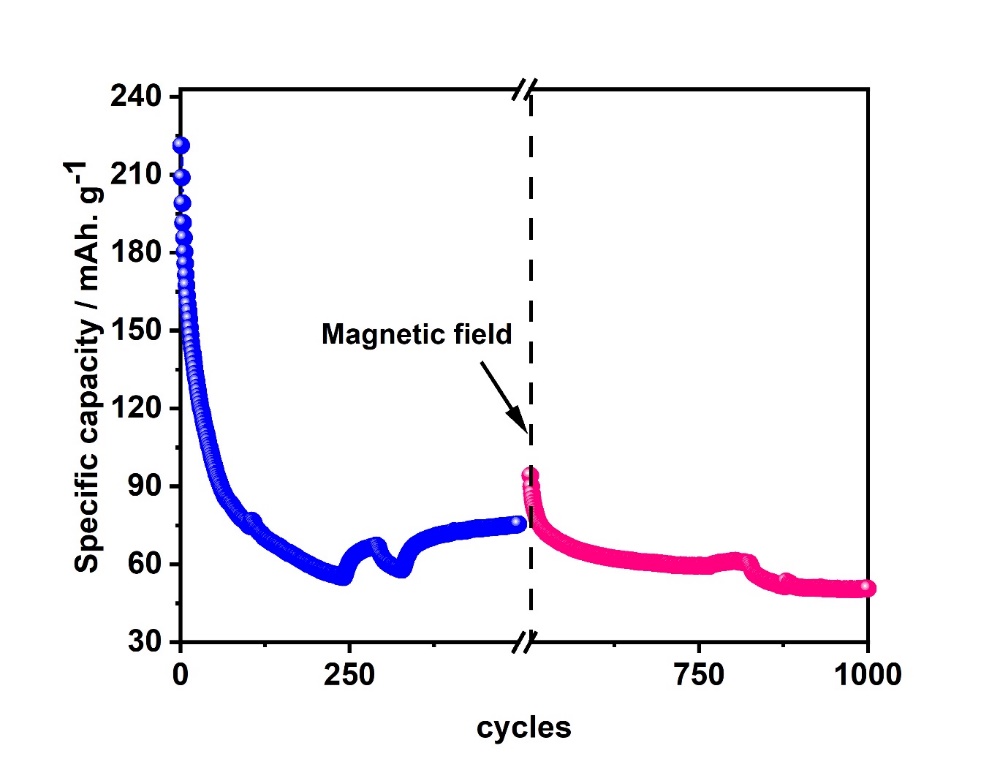
**

**Fig S16.** magnetic field applied at 501^st^ discharge cycle of α-Fe_2_O_3_/NC anode

**Cyclic stability of old cell**

Even the old cell can be revived in the presence of the magnetic field is shown in **Figure S17**. For cycled cells after long cycling capacity fade is observed due to the volume expansion and materials detachment. In the presence of magnetic field, it creates the shortest path for Li^+^ ions to reach anode and also creates stable SEI formation. Due to this, capacity gets increased, and stable charge-discharge profile is obtained for the cell for further cycles, and capacity also increases with cycling in presence of the magnetic field.


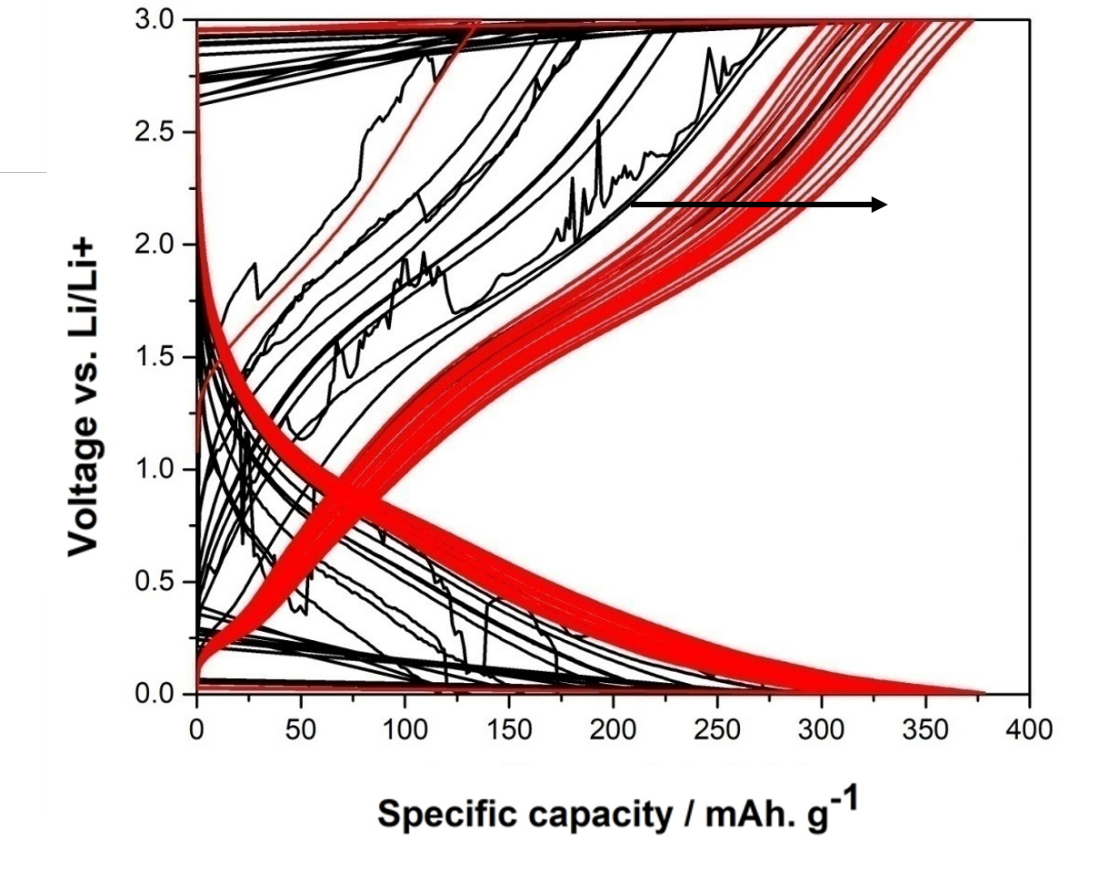


**Figure S17 .** Revived faded cell with magnetic field (black: in the absence of magnetic field, red: in presence of magnetic field)

**Charge-Discharge profile of commercial graphite:**

**Figure S18** shows the 1^st^, 100^th^ and 300^th^ charge discharge profile of commercial graphite at a current density of 1 A. g^-1^. After 300^th^ cycle discharge capacity has reduced from 279 mAh. g^-1^ to 150 mAh. g^-1^ as mentioned in the **Table S2**.


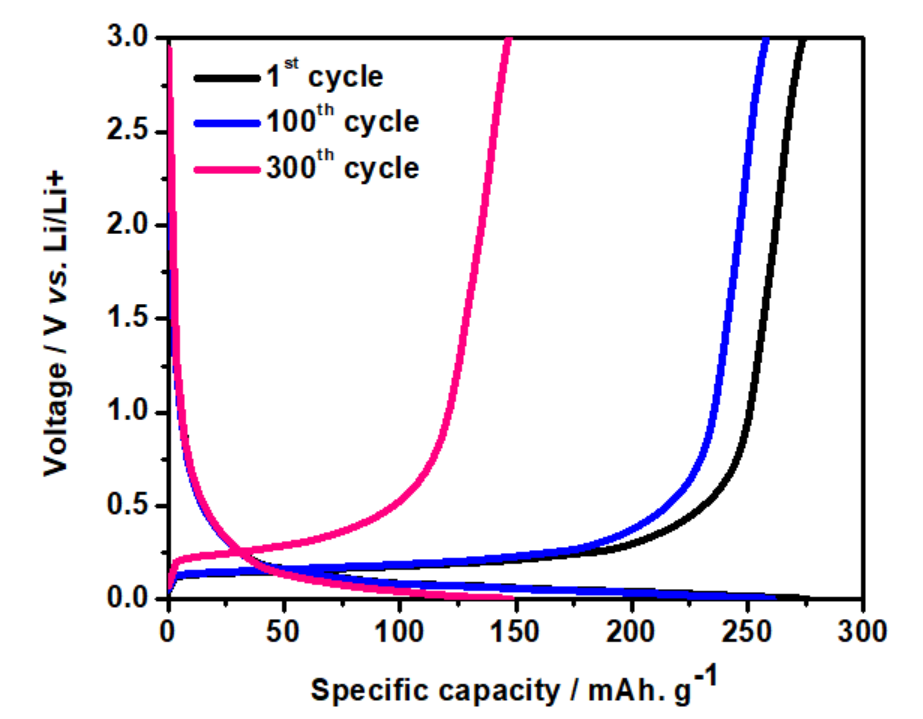


**Fig S18.** Charge- discharge profile of commercial graphite anode

**Characterization of paracetamol drug**

ATR-FTIR spectra of paracetamol drug is shown in **figure S19a**. Characteristic vibrational peaks for O-H and CH_3_ stretching are assigned at 3330 cm^-1^ and 3020-3200 cm^-1^ respectively. C=O and C=C stretching are attributed to vibrational peaks at 1650 cm^-1^ and 1610 cm^-1^. Amide (N-H) bending occurs at 1564 cm^-1^ and stretching occurred at 915 cm^-1^. Asymmetric C-H bending and C-C stretching occur at 1510 cm^-1^ and 1440-1435 cm^-1^, respectively. Absorption peaks at 1365-1330 cm^-1^ is assigned to C-H stretching and 1230-1265 cm^-1^ is attributed to C-N stretching. Also, peaks at 840 cm^-1^ is assigned to para substituted aromatic ring^12^.

Raman spectra of paracetamol drug in **figure S19 b** has revealed the Raman spectra of paracetamol dominated by the peaks at 797, 858 and 1235 cm^-1^ assigned to CNC stretching , ring breathing and C-C ring stretching respectively. 1324, 1560, 1611 and 1650 cm^-1^ are the characteristic amide III, amide II, ring stretching and amide I modes respectively^13^.

**Figures S19 (c-d)** show FESEM-EDAX images of paracetamol drug. **Figure S19 c** shows sheetlike morphology observed in FESEM images. EDAX confirms the presence of carbon, nitrogen, oxygen in the samples (**Figure S19 d**).


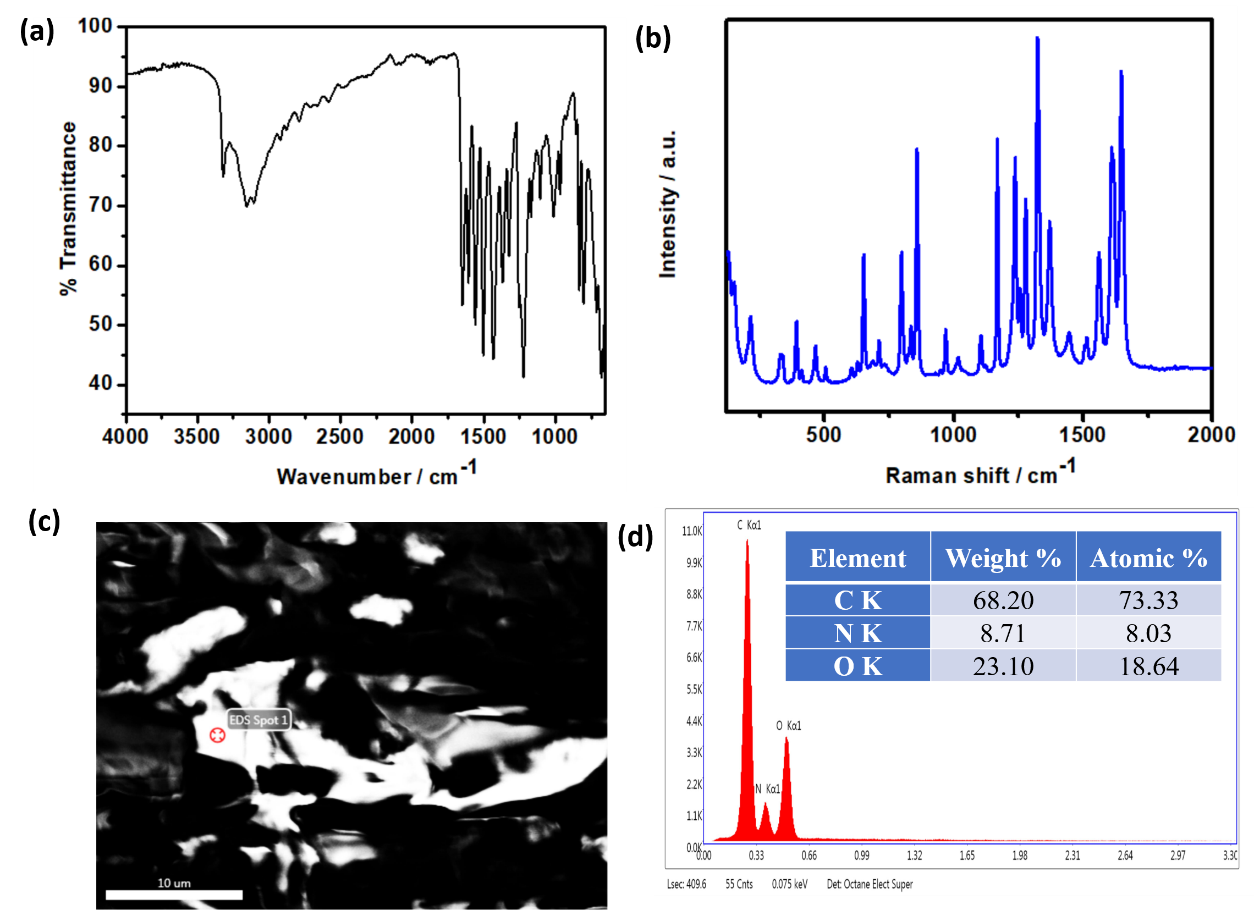


**Figure S19.** (a) ATR-FTIR spectra (b) Raman spectrum (c-d) FESEM-EDAX analysis of paracetamol drug used

1. **Debye-Scherrer formula**


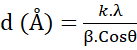
 (S1)

d = crystallite size,

λ = wavelength of X-ray

β = FWHM (in radian)

θ = diffraction angle

1. **Randles-sevcik equation**


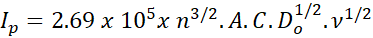
 (S2)

I_p_ = peak current (A)

D_o_= diffusion coefficient (cm^2^. s^-1^)

ν = scan rate (V. s^-1^)

n = number of charge transfer

A = effective surface area (m^2^. g^-1^)

C = concentration of lithium (mol. cm^-3^)

**Rietveld analysis of XRD data**

**Table S1** shows the 2θ values of commercial α-Fe_2_O_3_ and synthesised α-Fe_2_O_3_/NC . Shifting of all the peaks to lower angle indicates the strain induced due to the combustion reaction. Increasing d values indicates the reduced particle size of the material.

**Table S1**. Comparison of XRD of commercial α-Fe_2_O_3_ and synthesised α-Fe_2_O_3_/NC

| **α-Fe_2_O_3_/NC** | **Commercial**  **α-Fe_2_O_3_** | **Crystallographic Planes** | | |
| --- | --- | --- | --- | --- |
| **2θ / degrees** | | **h** | **k** | **l** |
| 23.67 | 24.128 | 0 | 1 | 2 |
| 32.650 | 33.118 | 1 | 0 | 4 |
| 35.145 | 35.612 | 1 | 1 | 0 |
| 40.517 | 40.829 | 1 | 1 | 3 |
| 49.025 | 49.419 | 0 | 2 | 4 |
| 53.629 | 54.004 | 1 | 1 | 6 |
| 57.039 | 57.412 | 1 | 2 | 2 |
| 62.032 | 62.386 | 2 | 1 | 4 |
| 63.697 | 63.964 | 0 | 3 | 0 |
| 71.521 | 71.822 | 1 | 0 | 10 |

**Table S2. Comparison of Li-ion battery anode materials with respect to specific capacity:**

| **Materials** | **Current density (A. g^-1^)** | **Specific capacity**  **(1^st^ cycle) (mAh. g^-1^)** | **Specific capacity**  **(300^th^ cycle) (mAh. g^-1^)** | **Capacity retention after 300 cycles** |
| --- | --- | --- | --- | --- |
| **Commercial Graphite** | 1 | 279 | 150 | 53.7% |
| ***α-Fe_2_O_3_-C (without Magnetic field)*** | 1 | 500 | 300 | 60% |
| ***α-Fe_2_O_3_-C***  ***(with 1^st^ cycle discharge-charge with magnetic field)*** | 1 | 800 | 550 | 68.75% |

**Table S3. Single charging with magnetic field for cycled cell (Magnetic field is applied at 501^st^, 1001^st^, 1501^st^, and 1751^st^ cycles)**

| **Anode Material** | **Current density** | **Specific capacity (mAh. g^-1^)** | | | | | | | | | | |
| --- | --- | --- | --- | --- | --- | --- | --- | --- | --- | --- | --- | --- |
|  |  | **1^st^ cycle** | **500^th^ cycle** | **501^st^ cycle** | **1000^th^ cycle** | **1001^st^ cycle** | **1500^th^ cycle** | **1501^st^ cycle** | **1700^th^ cycle** | **1751^st^ cycle** | | **2000^th^ cycle** |
| ***α-Fe_2_O_3_/NC*** | **5**  **A. g^-1^** | 220 | 80 | 150 | 120 | 130 | 110 | 100 | 100 | | 100 | 100 |
| ***% Capacity*** |  | 100 | 36.3 | 68.2 | 54.5 | 59.1 | 50 | 45.5 | 45.5 | 45.5 | | 45.5 |

References

1. Ganguly, D., Sundara, R. & Ramanujam, K. Chemical Vapor Deposition-Grown Nickel-Encapsulated N-Doped Carbon Nanotubes as a Highly Active Oxygen Reduction Reaction Catalyst without Direct Metal-Nitrogen Coordination. *ACS Omega* **3**, (2018).

2. Kraushofer, F. *et al.* Atomic-Scale Structure of the Hematite α-Fe2O3(11-02) ‘r-Cut’ Surface. *J. Phys. Chem. C* **122**, 1657–1669 (2018).

3. Grosvenor, A. P., Kobe, B. A., Biesinger, M. C. & McIntyre, N. S. Investigation of multiplet splitting of Fe 2p XPS spectra and bonding in iron compounds. *Surf. Interface Anal.* **36**, 1564–1574 (2004).

4. Mansour, H. *et al.* Structural, optical, magnetic and electrical properties of hematite (α-Fe2O3) nanoparticles synthesized by two methods: polyol and precipitation. *Appl. Phys. A Mater. Sci. Process.* **123**, 1–10 (2017).

5. Chmiel, F. P. *et al.* Observation of magnetic vortex pairs at room temperature in a planar α-Fe 2 O 3 /Co heterostructure. *Nat. Mater.* **17**, 581–585 (2018).

6. Bhowmik, R. N. & Saravanan, A. Surface magnetism, Morin transition, and magnetic dynamics in antiferromagnetic α-Fe2O3 (hematite) nanograins. *J. Appl. Phys.* **107**, (2010).

7. Brunauer, S., Emmett, P. H. & Teller, E. Adsorption of Gases in Multimolecular Layers. *J. Am. Chem. Soc.* **60**, 309–319 (1938).

8. Zhang, Q. *et al.* Lithium-Ion Battery Cycling for Magnetism Control. *Nano Lett.* **16**, 583–587 (2016).

9. Ngamchuea, K., Tschulik, K. & Compton, R. G. Magnetic control: Switchable ultrahigh magnetic gradients at Fe<inf>3</inf>O<inf>4</inf> nanoparticles to enhance solution-phase mass transport. *Nano Res.* **8**, 3293–3306 (2015).

10. Mutschke, G. & Bund, A. On the 3D character of the magnetohydrodynamic effect during metal electrodeposition in cuboid cells. *Electrochem. commun.* **10**, 597–601 (2008).

11. Mutschke, G. *et al.* On the action of magnetic gradient forces in micro-structured copper deposition. *Electrochim. Acta* **55**, 9060–9066 (2010).

12. Castro-Suarez, J. R., Vásquez-Osorio, M., Hernandez-Rivera, S. P. & Pájaro-Payares, A. A. Vibrational analysis of acetaminophen from commercial tablets. *IOP Conf. Ser. Mater. Sci. Eng.* **519**, (2019).

13. Shende, C., Smith, W., Brouillette, C. & Farquharson, S. Drug stability analysis by Raman spectroscopy. *Pharmaceutics* **6**, 651–662 (2014).
